# Cortical circuitry mediating interareal touch signal amplification

**DOI:** 10.1016/j.celrep.2023.113532

**Published:** 2023-12-06

**Authors:** Lauren Ryan, Andrew Sun-Yan, Maya Laughton, Simon Peron

**Affiliations:** 1 Center for Neural Science, New York University, 4 Washington Place, Rm. 621, New York, NY 10003, USA; 2 Lead contact

## Abstract

Sensory cortical areas are organized into topographic maps representing the sensory epithelium. Interareal projections typically connect topographically matched subregions across areas. Because matched subregions process the same stimulus, their interaction is central to many computations. Here, we ask how topographically matched subregions of primary and secondary vibrissal somatosensory cortices (vS1 and vS2) interact during active touch. Volumetric calcium imaging in mice palpating an object with two whiskers revealed a sparse population of highly responsive, broadly tuned touch neurons especially pronounced in layer 2 of both areas. These rare neurons exhibited elevated synchrony and carried most touch-evoked activity in both directions. Lesioning the subregion of either area responding to the spared whiskers degraded touch responses in the unlesioned area, with whisker-specific vS1 lesions degrading whisker-specific vS2 touch responses. Thus, a sparse population of broadly tuned touch neurons dominates vS1-vS2 communication in both directions, and topographically matched vS1 and vS2 subregions recurrently amplify whisker touch activity.

## In brief

Ryan et al. show that a sparse group of broadly tuned touch neurons dominates communication between primary and secondary vibrissal somatosensory cortices in mice. Subregions of these areas that respond to the same whiskers work to mutually amplify the overall cortical response to touch.

## Graphical abstract

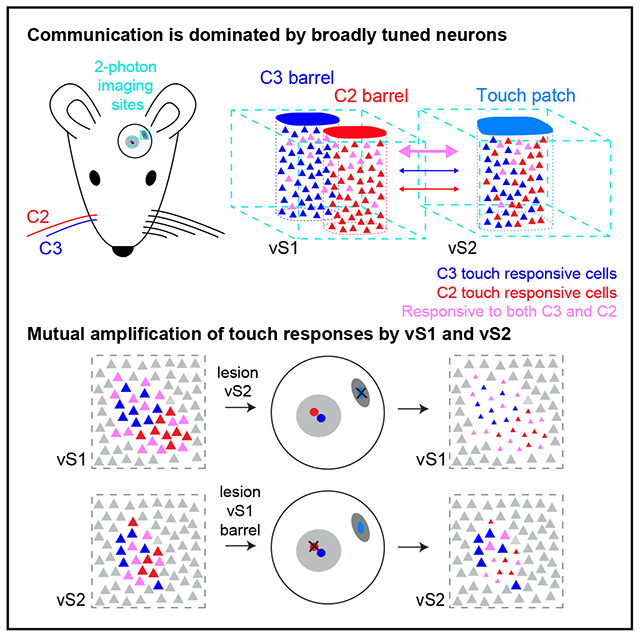

## INTRODUCTION

Sensory cortices of a given modality are richly interconnected,^1^ and in many cases, reciprocal projections preferentially link cortical subregions responding to the same portion of the sensory epithelium.^2,3^ What is the role of such topography-respecting circuitry? In the canonical columnar microcircuit,^4^ thalamic input strongly activates cortical layer (L) 4,^5,6^ which drives activity in L2/3.^7^ Within L2/3, similarly tuned neurons exhibit elevated recurrent connectivity,^8^ which enables pattern completion^9^ and amplification.^10,11^ L2/3 of many cortical areas outputs extensively to^12,13^ and receives input from^14^ other cortical areas, particularly adjacent ones.^15^ It is thus likely that local recurrent processing in L2/3 is complemented by interareal recurrence mediated by topography-respecting projections.

Topographically matched recurrent amplification between cortical areas may be a defining characteristic of sensory cortex and could facilitate coordination across subregions of cortex that respond to the same stimulus. This coordination may be important for many computations involved in perception. Such recurrence could drive the location-specific response enhancement that is characteristic of spatial attention,^16,17^ undergird feature binding across areas,^16,18^ and facilitate object recognition by ensuring relatively synchronous activation of cortical subregions responding to the same stimulus across distinct cortical areas.^19,20^

In the mouse vibrissal system, thalamic input from individual whiskers terminates on a small patch of vibrissal S1 (vS1) called a “barrel.”^21^ Vibrissal S2 (vS2) also shows somatotopically organized responses to whisker touch but with a more compressed map.^22,23^ Vibrissal S1 and S2 are extensively interconnected in a topography-respecting manner^2^ and respond robustly to whisker touch.^23,24^ Axonal projections both from vS1 to vS2 and from vS2 to vS1 carry strong touch signals.^2,14,22,25^ This suggests that somatotopically matched (“iso-somatotopic”) subregions of vS1 and vS2 may recurrently enhance one another’s responses to whisker touch.^22^

How do iso-somatotopic subregions of vS1 and vS2 interact during active touch? Here, we focus on the subregions of vS1 and vS2 that respond to touch from whiskers C2 and C3. We first employ volumetric two-photon calcium imaging^26^ of iso-somatotopic subregions of vS1 and vS2 to characterize touch neuron populations in both areas. Next, we use retrograde labeling to determine which populations of touch neurons relay touch information to iso-somatotopic targets across both areas. Finally, we selectively lesion subregions of either vS1 or vS2 responsive to touch by one or both spared whiskers to assess how iso-somatotopic sites mutually influence one another.

## RESULTS

### Mapping neural activity in vS1 and vS2 during an active touch task

**Figure 1.**
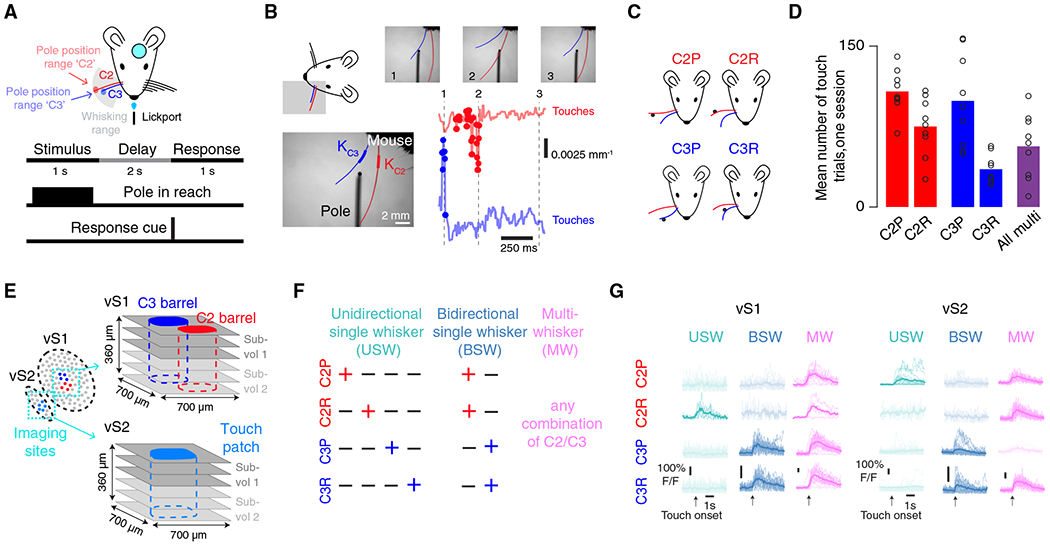
Volumetric two-photon imaging in vS1 and vS2 during an active two-whisker touch task (A) Experimental setup. Head-fixed mice palpated poles that were presented within range of one of their two spared whiskers on each trial. Mice received a reward on a random 70% of trials. Bottom, task timing. On each trial, a pole was presented for 1 s and was then removed. Following a 2-s delay, which ended with a response cue, mice had 1 s to respond by licking. (B) Whisker videography. Bottom left, example frame showing traced whiskers and where curvature of each whisker is measured. Scale bar, 2 mm. Top right, three example frames that correspond to points in time on the Δκ traces below. Bottom right, Δκ trace for each whisker, with touches overlaid. (C) Four types of single whisker touch: whisker C2 protraction (C2P), whisker C2 retraction (C2R), whisker C3 protraction (C3P), and whisker C3 retraction (C3R). (D) Number of trials of a given touch type over one imaging session, n = 9 mice. Bars, mean; circles, individual mice. Red, whisker C2 touch trials; blue, whisker C3 touch trials; purple, dual-whisker touch trials. (E) Volumetric calcium imaging. Left, placement of cranial window allows for imaging of both vS1 and vS2. Subregions responsive to spared whiskers are colored. Right, groups of three planes of the same shade constitute a simultaneously imaged subvolume. (F) Touch cell type classification. Each column represents an example cell of the shown category, with plus indicating that the neuron responds to that touch type. (G) Example ΔF/F traces for each cell type from each area (left, vS1; right, vS2) for one example session from one animal. Thin lines, individual trial responses; thick line, mean across trials.

We implanted transgenic mice expressing GCaMP6s^27^ in cortical excitatory neurons (Ai162 x Slc17a7-Cre)^28^ with a cranial window over vS1 and vS2 (STAR Methods). Following recovery, mice were trimmed to two whiskers (C2 and C3) and trained on a head-fixed active whisker touch task. Each trial started with a 1-s stimulus period during which a pole became accessible to palpation near the tip of one of the two spared whiskers (Figure 1A). The pole was then withdrawn, and a 2-s delay period began. Finally, a tone indicated the start of a 1-s response period, during which licking resulted in a water reward on a random 70% of trials. This task design ensured that animals whisked actively and were rewar d motivated, both of which influence sensory cortical responses.^29,30^

We employed high-speed whisker videography to capture whisker movement (Figure 1B; 400 Hz; STAR Methods). Whisker video was segmented to track individual whiskers^31^ (STAR Methods), allowing us to measure changes in whisker curvature (Δκ), which was used as a proxy for force at the whisker follicle.^32^ We divided touches into four types (Figure 1C): protractions of whisker C2 (C2P), protractions of whisker C3 (C3P), retractions of whisker C2 (C2R), and retractions of whisker C3 (C3R). We used a range of pole positions to ensure many isolated touches of each type (Figures 1A and 1D), with most trials containing touch (79.4% ± 4.4% trials with touch, mean ± SD, n = 9 mice).

In trained mice (n = 9; Table S1) with consistent whisking, we recorded activity during the task using volumetric two-photon calcium imaging^26^ (Figure 1E). We first determined the precise locations in both vS1 and vS2 that responded to touch by the two spared whiskers by passively deflecting the whiskers during widefield two-photon calcium imaging of these areas (STAR Methods; Figure S1). In vS1, we found the locations corresponding to the C2 and C3 barrels. In vS2, we located the patch of touch cells that responded to touch from either C2, C3, or both. In both areas, we performed cellular-resolution recordings centered on these touch-responsive subregions, simultaneously imaging three individual planes (700 by 700 μm) spaced 60 μm apart in depth (STAR Methods; 7 Hz). These planes comprised a “subvolume,” and we imaged two subvolumes per area (Table S1), starting at the L1-L2 boundary. While all subvolumes were visited in one session, only one subvolume was imaged at a time (STAR Methods). We recorded across all or most of L2/3 depth-wise in both areas (8,864 ± 563 neurons per mouse, n = 9 mice).

We classified neurons as responsive to a particular touch type if they responded on at least 5% of trials that contained only that touch type (STAR Methods). Neurons that responded to at least one touch type were considered touch neurons and were further classified based on the specific touch type(s) they responded to. Neurons that responded to only one whisker touching in a specific direction were classified as unidirectional single-whisker (USW) cells; neurons that responded to both protraction and retraction touches for only one whisker were classified as bidirectional single-whisker (BSW) cells; neurons that responded to any combination of C2 and C3 touches were classified as multiwhisker (MW) cells^33^ (Figure 1F). We found neurons of each type in both vS1 and vS2 (Figure 1G).

### Multiwhisker cells are rare but respond robustly to touch in both vS1 and vS2

**Figure 2.**
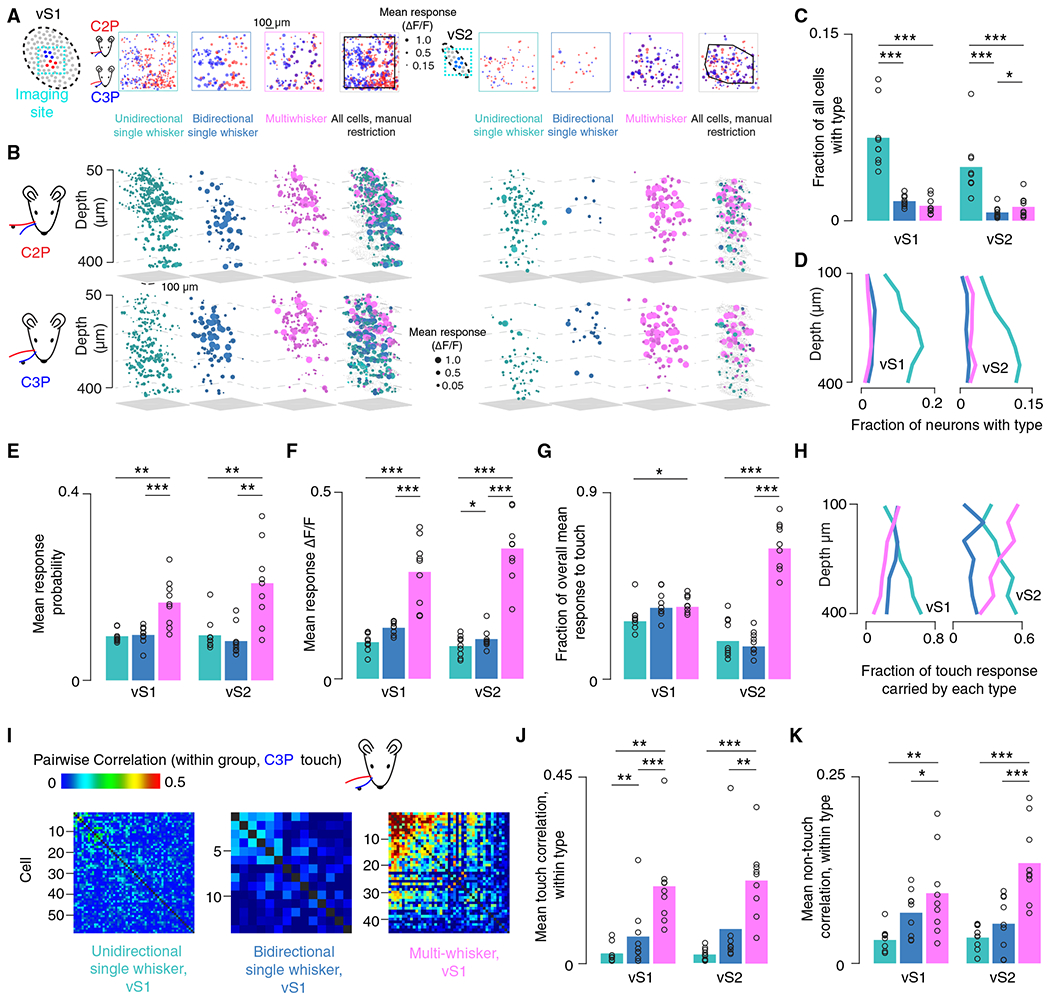
A sparse population of multiwhisker cells exhibits robust touch responses in superficial vS1 and vS2 (A) Projection across depth of mean touch-evoked ΔF/F to C2P (red) and C3P (blue) touches in vS1 and vS2 imaging sites in an example animal. Only cells with mean touch-evoked ΔF/F greater than 0.15 are included. Left to right: unidirectional single whisker neurons; bidirectional single whisker neurons; multiwhisker neurons; all neurons, with manual restriction border shown in black (STAR Methods). Left four panels, vS1; right four panels, vS2. (B) Example neurons from (A) shown in 3D. Top, mean touch-evoked ΔF/F following C2P touch; bottom, C3P touch. Left to right, mean touch-evoked ΔF/F among unidirectional single whisker neurons (cyan), bidirectional single whisker neurons (dark blue), multiwhisker neurons (magenta), and all neurons in sub-selected region (gray dots represent cells not responsive to touch). Left four panels, vS1; right four panels, vS2. (C) Frequency of each of the major touch neuron types in both areas. Bars, mean (n = 9 mice). p values indicated for two-tailed paired (paired within animal) t tests: *p < 0.05, **p < 0.01, ***p < 0.001. (D) Fraction of neurons belonging to type at given depth (bin size: 50 μm) in each area. (E) Mean response probability for each type in each area. For neurons responsive to more than one touch type (BSW, MW), mean response probability was averaged across all touch types for which individual neurons were deemed responsive. (F) Mean touch-evoked ΔF/F averaged across touch types for which individual neurons were deemed responsive. (G) Fraction of the overall mean ΔF/F response to touch in each area that is contributed by each population. (H) Fraction of touch response carried by each type at each depth (bin size: 50 μm). (I) Example within-type C3P touch-evoked correlation matrices for each touch cell type in vS1 in an example animal (STAR Methods). (J) Mean within-type pairwise correlations in each area for periods of touch. (K) Same as in (J), but “spontaneous” correlations for periods of non-touch.

In superficial vS1, broader tuning and sparser responses are especially pronounced in L2.^33,34^ To determine if a similar organization was present in vS2, we examined the touch neuron distribution in vS1 and vS2, finding a mix of all three touch types in both areas (Figures 2A and 2B). We imaged vS1 and vS2 in 9 mice across a depth of 360 μm starting at the L1-L2 boundary. Because the vS2 topographic map is compressed in comparison to the map in vS1, we sub-selected an approximately 300- by 600-μm area centered on the most touch-responsive neurons, yielding 1,963 ± 408 vS2 neurons (STAR Methods; mean ± SD, n = 9 mice). This subregion contained the majority of C2- and C3-responsive neurons. For vS1, we used a 600- by 600-μm area centered on the barrels of the two spared whiskers (neuron count: 4,017 ± 607).

Vibrissal S2 contained a smaller fraction of USW (Figure 2C; USW fraction, vS1: 0.067 ± 0.026, vS2: 0.044 ± 0.025, n = 9 mice, p = 0.008, within animal paired t test comparing vS1 and vS2) and BSW cells (BSW fraction, vS1: 0.016 ± 0.005, vS2: 0.007 ± 0.005, p = 0.004) than vS1, with a comparable number of MW neurons (MW fraction, vS1: 0.012 ± 0.007, vS2: 0.011 ± 0.009, p = 0.820). Both areas had substantially more USW neurons than BSW or MW neurons (vS1, USW vs. BSW, p < 0.001; USW vs. MW, p < 0.001; vS2, USW vs. BSW, p < 0.001; USW vs. MW, p < 0.001). In vS2, MW cells were more frequent than BSW cells (BSW vs. MW, p = 0.015); in vS1, this was reversed, though the difference was not significant (p = 0.931). Broadly tuned neurons (i.e., MW and BSW neurons) thus made up a small fraction of neurons in both areas.

We next determined the depth distribution of the different touch populations starting at the L1-L2 border. All three touch neuron types declined in frequency from deep L3 to superficial L2 in both areas, with broadly tuned neurons declining more slowly (Figure 2D). We then restricted our analyses to the most superficial three imaging planes where these broadly tuned neurons were relatively more numerous and asked which population contributed the most to the touch response. In both areas, the MW neurons had a significantly higher touch response probability compared to BSW (Figure 2E, n = 9, vS1: p < 0.001, within animal paired t test; S.2: p = 0.002) and USW cells (vS1: p = 0.003; vS2: p = 0.003). MW neurons in both areas also had a significantly larger mean touch-evoked ΔF/F response (averaged only across touch types to which a neuron was considered responsive; STAR Methods) than BSW (Figure 2F, n = 9 mice, vS1: p < 0.001, vS2: p < 0.001) and USW cells (vS1: p < 0.001, vS2: p < 0.001). Therefore, in superficial L2/3, MW neurons exhibit the strongest touch responses. These trends persisted when including neurons from all depths (Figure S2).

Does the high responsiveness of MW neurons compensate for their rarity, resulting in this population carrying much of the touch response within vS1 and vS2? To address this, we computed the mean touch-evoked ΔF/F for every touch neuron for each individual touch. We then summed across all neurons of a given touch type and divided each group’s net response by the net response across all neurons, resulting in an estimate of the fractional contribution each population made for every touch. We finally computed the mean contribution of each population across all touches (STAR Methods). In vS1, we found that each group carries a relatively comparable proportion of the touch response (Figure 2G). In vS2, however, MW neurons were responsible for most of the overall response to touch (MW vs. USW: p < 0.001, MW vs. BSW: p < 0.001). Therefore, despite their rarity, MW neurons carry a disproportionately large fraction of the touch response in superficial L2/3 in both areas and comprise the majority of the vS2 touch response.

We next asked what fraction of the touch response is carried by each population across depth. Broadly tuned MW neurons contribute an increasing fraction of the touch response superficially in both areas, with the response of MW neurons becoming especially dominant in superficial vS2 (Figure 2H). In contrast, USW and BSW cells showed a declining fractional response in both areas superficially. As input from L4 is transformed by local circuitry on its way to L2,^35^ the touch response thus becomes increasingly concentrated among MW neurons in both areas.

If MW neurons exhibit elevated synchrony relative to other touch populations, they would be more effective at driving putative downstream targets.^36^ We therefore compared touch-evoked correlations between neurons within each population (Figure 2I; STAR Methods). MW cells exhibited significantly higher touch-evoked correlations than other populations (Figure 2J; n = 9 mice, vS1: MW vs. USW p = 0.003, MW vs. BSW p < 0.001, vS2: MW vs. USW p ≤ 0.001, MW vs. BSW p = 0.003). This pattern was also present in “spontaneous correlations” measured during the non-touch epoch (STAR Methods): MW cells were significantly more correlated to one another compared with other touch cell types (Figure 2K, n = 9 mice, vS1: MW vs. USW p = 0.008, MW vs. BSW p = 0.043, vS2: MW vs. USW p < 0.001, MW vs. BSW p < 0.001). Thus, in addition to their larger touch responses, MW cells exhibit greater synchrony, making them especially well-suited to influence downstream populations. Elevated spontaneous correlations suggest that this synchrony may be due to elevated connectivity among these neurons.^8^

In sum, despite MW cells constituting a minority of touch-responsive neurons, these cells responded to touch strongly, reliably, and with elevated synchrony in both vS1 and vS2. These neurons thus play a disproportionate role in the superficial touch response in both areas.

### Multiwhisker neurons contribute disproportionately to the vS1-vS2 projection touch response

**Figure 3.**
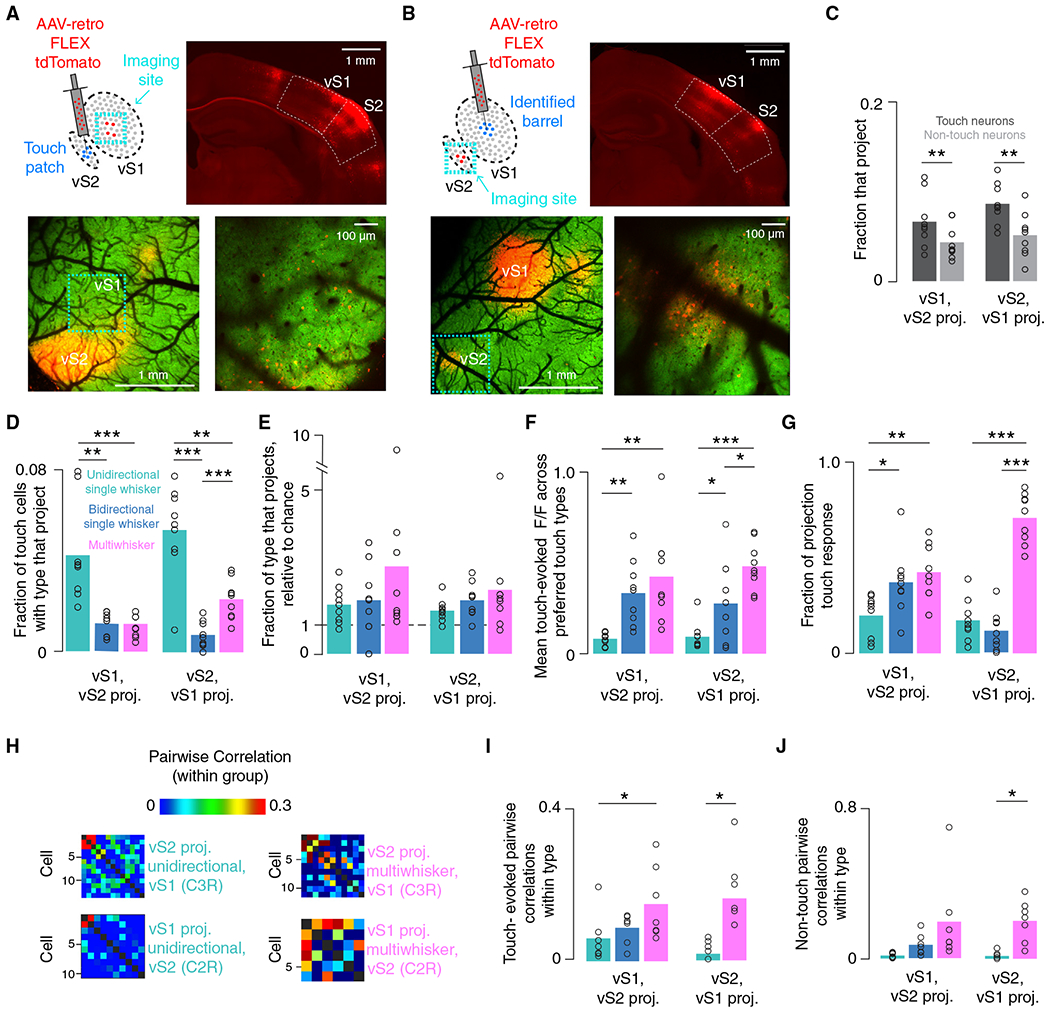
Multiwhisker cells carry a large fraction of the vS1-vS2 projection touch response in both directions (A) Retrograde injection into vS2. Top right, coronal section showing injection in vS2 and retrograde viral tdTomato expression in vS1. Scale bar, 1 mm. Bottom left, widefield two-photon image showing labeled cell expression. Green, GCaMP6s; red, tdTomato. Cellular-resolution imaging site is outlined in cyan. Scale bar, 1 mm. Bottom right, example cellular-resolution two-photon imaging plane in vS1 showing cells expressing GCamp6s and tdTomato. Scale bar, 100 μm. Injection is made into a touch patch (in L2/3) identified by cellular-resolution imaging prior to injection (STAR Methods). (B) As in (A) but for vS2 imaging following vS1 injection. Injection is made into single barrel (L2/3) identified by cellular-resolution imaging prior to injection (STAR Methods). (C) Fraction of touch (darkgray) and non-touch (light gray) neurons that project from each are a to the other. Bars, mean (n = 9 mice); circles, individual animals. p values indicated for two-tailed paired (paired within animal) t tests: *p < 0.05, **p < 0.01, ***p < 0.001. (D) Fraction of touch cells of given type that project, two-sample t test. (E) Fraction of each touch subpopulation that projects to the other area, normalized to fraction of all neurons belonging to that subpopulation (“chance”). (F) Mean ΔF/F in response to touch averaged across touch types for which individual projecting neurons are responsive. (G) Fraction of overall touch-evoked ΔF/F (STAR Methods) among projecting neurons that is contributed by each type in both areas. (H) Example within-type C3R touch correlation matrix for two example populations in one mouse that project from vS1 to vS2 and C2R responses for analogous populations in a second mouse projecting from vS2 to vS1. (I) Mean within-type pairwise correlations during the period around touch (n = 7 mice; STAR Methods). (J) Mean within-type pairwise correlations during non-touch “spontaneous” period for each touch population in each area (STAR Methods).

Vibrissal S1 and S2 are richly interconnected^2,22,37,38^ and respond robustly to whisker touch.^2,23,39^ Do broadly tuned touch neurons contribute disproportionately to the touch signal carried between iso-somatotopic vS1 and vS2 subregions? We labeled projecting neurons in both areas by focally injecting rAAV2-*retro*-FLEX-tdTomato into L2/3 of either vS1 or vS2^40^ (Figures 3A and 3B). We targeted either the center of the spared barrels in vS1 or the center of the “touch patch” responding to the spared whiskers in vS2 (STAR Methods). Injections into the vS2 touch patch led to a diffuse labeling across vS1 (Figures 3A and S3), implying that vS1 sends broad touch input to vS2. In contrast, vS1 injections led to focal expression in the vS2 touch patch (Figure 3B). Vibrissal S2 therefore sends more spatially confined input to vS1.

We imaged the subregion of the uninjected area that responded to touch by the two spared whiskers. In both areas, we imaged two three-plane subvolumes spanning 90–120 μm each (total: 180–240 μm in depth; Table S1), restricting analysis to an approximately 600- by 600-μm patch in vS1 and an approximately 300- by 600-μm patch in vS2 (STAR Methods). We found 180 ± 49 vS2-projecting neurons in vS1 out of a total 4,109 ± 788 neurons (n = 9 mice) and 97 ± 58 vS1-projecting neurons in vS2 out of 1,705 ± 550 (n = 9 mice) neurons. In both areas, touch neurons were more likely to project to the other area than non-touch neurons (Figure 3C): in vS1, 7.0% ± 3.0% of touch neurons projected to vS2, whereas only 4.0% ± 2.0% of non-touch neurons projected to vS2 (within animal paired t test, touch vs. non-touch p = 0.003). In vS2, 9.0% ± 2.0% of touch neurons and 5.0% ± 2.0% of non-touch neurons projected to vS1 (p = 0.002). Thus, touch neurons are more likely to project in both directions compared to non-touch neurons.

We next examined the composition of the projecting populations. Among vS2-projecting neurons in vS1, USW neurons were more numerous than BSW and MW neurons (Figure 3D; two-sample t test, n = 9 mice, USW vs. BSW p = 0.001; USW vs. MW p < 0.001). A similar pattern held in vS2 (n = 9 mice, USW vs. BSW p < 0.001; USW vs. MW p = 0.001). In vS2, MW neurons were more likely to project than BSW neurons (p < 0.001). Given that USW neurons were more numerous in both areas (Figure 2C), we asked if specific touch cell types projected more than expected by chance. We divided the fraction of the projecting population consisting of a particular type by the fraction of the overall population consisting of that type. This number exceeded 1 for all touch types (Figure 3E), implying that all touch neuron types projected in both directions more than predicted by their frequency.

Did touch-evoked response amplitude differ across specific projecting touch populations? Among vS1 neurons projecting to vS2, MW cells responded more strongly on average than USW cells (Figure 3F, n = 9 mice, USW vs. MW p = 0.001, two-sample t test), as did BSW cells (USW vs. BSW, p = 0.001). Among vS2 neurons projecting to vS1, MW cells again responded more strongly on average than USW cells (n = 9 mice, USW vs. MW p < 0.001), as did BSW cells (USW vs. BSW, p = 0.040). MW projecting neurons also responded more strongly on average compared to BSW neurons in vS2 (BSW vs. MW, p = 0.037). MW neurons thus exhibit the largest touch responses among projecting neurons, particularly in vS2.

What fraction of touch-evoked activity among projecting neurons did specific populations contribute? For vS1 neurons projecting to vS2, MW neurons carried the largest fraction of the touch response (Figure 3G 0.43 ± 0.14, n = 9 mice), significantly more than the fraction carried by USW neurons (0.20 ± 0.11, p = 0.002, two-sample t test comparing MW vs. USW fraction). BSW neurons also carried a larger fraction of the touch response than unidirectional neurons (0.37 ± 0.17, p = 0.020, t test comparing USW vs. BSW fraction). Among vS2 neurons projecting to vS1, MW neurons carried the largest fraction of the touch response (MW: 0.71 ± 0.13; BSW: 0.11 ± 0.10; USW: 0.17 ± 0.11; MW vs. USW, p < 0.001; MW vs. BSW, p < 0.001). Despite their rarity, MW neurons carry the largest fraction of the touch response for both projections. Consequently, MW cells may be responsible for coordinating the output of the touch response in each area.

If MW projecting neurons are indeed coordinating and relaying the touch response of an area, we would also expect these neurons to exhibit a high level of synchrony, as this would make them potentially more effective at influencing downstream activity.^36^ We examined touch-evoked correlations in specific populations of projecting neurons (STAR Methods; only some animals had enough projecting neurons to calculate correlations). Among vS1 neurons projecting to vS2, MW projecting neurons had higher within-group pairwise correlations than USW neurons near the time of touch (Figures 3H and 3I MW vs. USW, p = 0.021, within animal paired t test, n = 7). Due to a paucity of projecting BSW cells in vS2, we only examined USW and MW neurons in vS2, with MW neurons exhibiting higher correlations around the time of touch (MW vs. USW, p = 0.046, n = 7). We found no significant differences in the touch-evoked pairwise correlations between projecting and non-projecting neurons near the time of touch. In both vS1 and vS2, MW projecting neurons had higher spontaneous correlations outside the touch epoch, though only the difference between MW and USW cells was significant (Figure 3J; MW vs. USW, p = 0.025, n = 7). Projecting MW neurons are thus highly correlated both during touch and non-touch epochs and thus more likely to send a synchronized signal downstream.

Overall, broadly tuned neurons make up a disproportionate fraction of projecting neurons across iso-somatotopic sites in vS1 and vS2. Projecting MW cells exhibited elevated synchrony, suggesting that they evoke downstream activity more effectively.

### Focal vS2 lesions degrade vS1 touch responses

**Figure 4.**
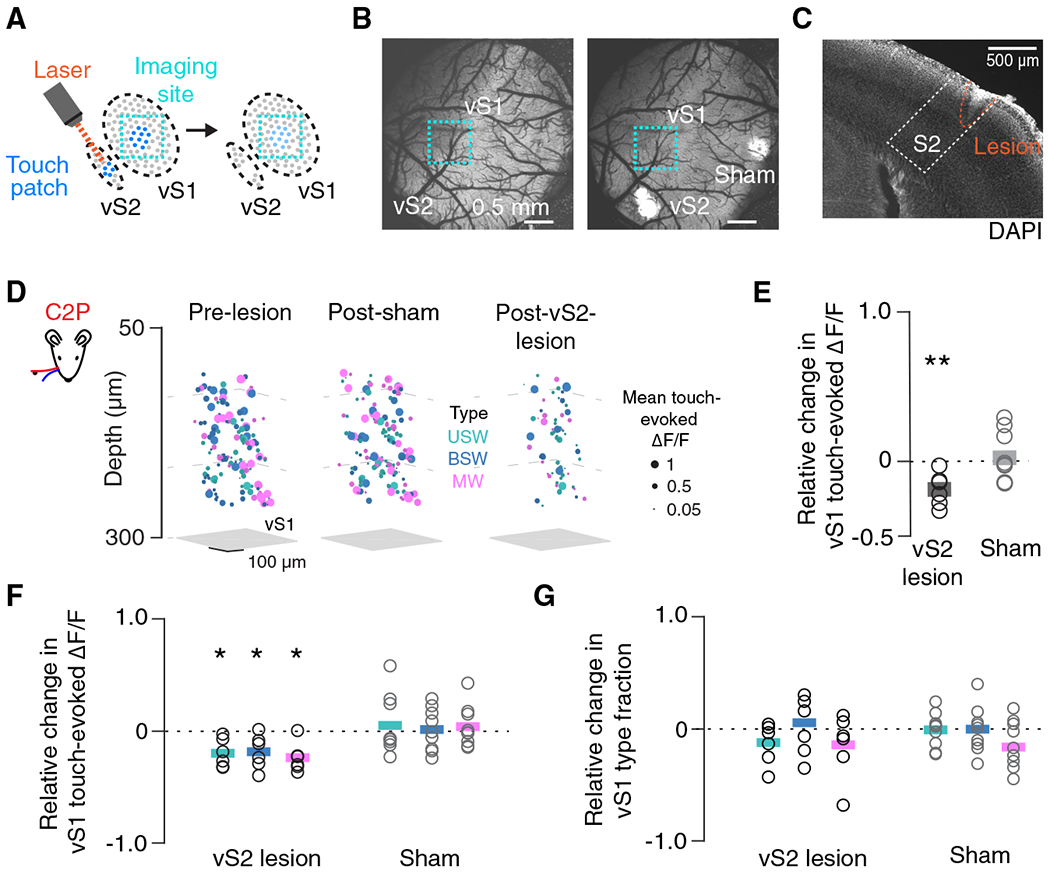
Touch response in vS1 declines after focal vS2 lesion (A) Lesion targeting the touch patch in vS2. (B) Wide field two-photon image (4x, STAR Methods) depicting the brain before and after lesions in an example animal. Vibrissal S2 lesion took place 24 h prior to right image; sham lesion took place 72 h prior to vS2 lesion. Scale bar, 0.5 mm. (C) Coronal section showing vS2 lesion from an example animal, with S2 outline based on Allen Brain Atlas alignment via SHARP-track registration (STAR Methods). Scale bar, 500 μm. (D) Mean touch-evoked ΔF/F to whisker C2 protractions for all responsive neurons in an example mouse’s vS1 with neurons colored by touch type. Non-responsive neurons are excluded. Left, prior to any lesion. Middle, session after sham lesion. Right, session after vS2 lesion. (E) Relative change in touch-evoked ΔF/F averaged across touch types in vS1 before and after vS2 lesion. Bar, mean (n = 6 mice). Gray, sham condition (n = 9 mice). p values indicated for two-sided t test of equality with 0 (dotted line): *p < 0.05, **p < 0.01. (F) As in (E) but broken up by touch type subpopulation. (G) As in (F) but for relative change in touch type fraction in vS1 before and after vS2 lesion. No population showed a significant change (p < 0.05) in fraction.

Given that the touch responses of MW neurons in vS2 contribute disproportionately to the vS1 projection, we next asked how vS2 touch activity contributes to iso-somatotopic touch responses in vS1. We ablated the vS2 touch patch using prolonged exposure to a femtosecond laser source^41^ (STAR Methods), resulting in a focal, superficial vS2 lesion (Figures 4A–4C). Before and after vS2 lesions, we imaged vS1 using a single three-plane subvolume spanning 180 mm in total depth (Table S1). We examined the impact of vS2 lesions on touch responses in six mice across 2,477 ± 465 vS1 neurons confined to a ~600- by 600-μm subregion (Figure 4D). Lesions significantly reduced the relative mean touch-evoked ΔF/F in vS1 (Figure 4E; p = 0.009, n = 6 mice, t test comparing relative change to 0). We found the same relative decline in touch-evoked ΔF/F among all three touch cell types (Figure 4F; USW, p = 0.013; BSW, p = 0.031; MW, p = 0.011). Though the response magnitude declined in all three populations, there was no change in the fraction of each touch cell type in vS1 after vS2 lesion (Figure 4G; USW, p = 0.189; BSW, p = 0.745; MW, p = 0.324), indicating that the touch cells are robust in identity, and that the reduced responsiveness is not due to a decrease in the proportion of touch cells. Vibrissal S2 thus augments the vS1 response to touch by providing broad touch input to all touch cell types.

To control for nonspecific effects, we preceded vS2 lesions with a “sham” lesion of a non-vibrissal area (Figure 4B). Following sham lesions, touch-evoked ΔF/F in vS1 remained unchanged (Figure 4E, p = 0.704, n = 9 mice, t test comparing relative change to 0). Sham lesions did not change the responsiveness of specific touch populations (Figure 4F; USW, p = 0.592; BSW, p = 0.850; MW, p = 0.550). Sham lesions also did not affect the fraction of touch cells of each type present in vS1 (Figure 4G; USW, p = 0.866; BSW, p = 0.955; MW, p = 0.103). We next asked whether vS2 lesions impacted vibrissal kinematics, as reduced touch intensity could account for the reduced response to touch. Touch count, the peak curvature during touch, and the peak velocity of whisking remained unchanged following vS2 lesions (Figure S4). We also did not find an overall change in correlation structure in vS1 after lesioning (Figure S5), suggesting that the pattern of touch-evoked activity in vS1 is not altered but simply reduced in intensity. Finally, we looked at the whisker movement-responsive cells (“whisking” neurons; STAR Methods) and found that they do not decrease in number or responsiveness after lesions (Figure S6). Thus, the lesion effect is specific to the touch-responsive population. Neurons of a given touch type transition to different touch and non-touch types even without perturbation; lesions did not alter these dynamics (Figure S7).

In sum, vS2 lesions result in a general decline in touch-evoked responsiveness in iso-somatotopic vS1. Nevertheless, neurons remain responsive to touch, and the local correlation structure remains unaltered. Vibrissal S2 therefore non-selectively enhances the touch response in iso-somatotopic vS1.

### Focal vS1 lesions degrade vS2 touch responses in a whisker-specific manner

**Figure 5.**
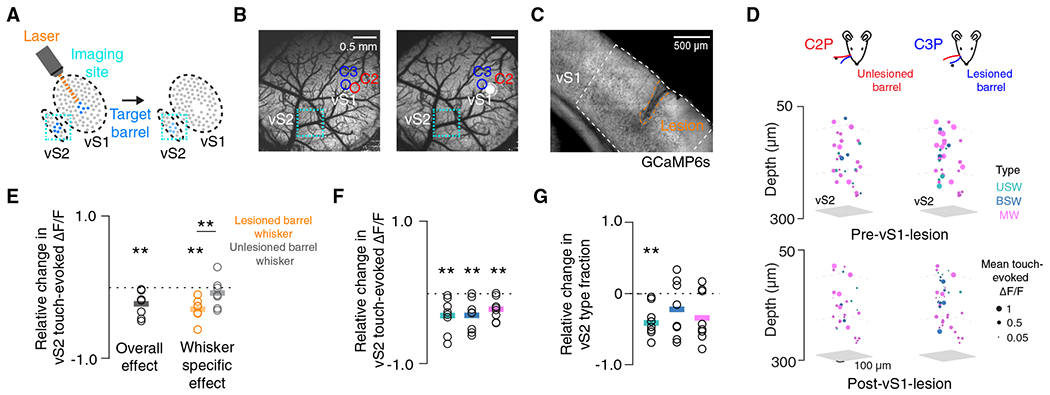
Touch response in vS2 declines in a whisker-specific manner after vS1 focal lesion (A) Lesion targeting single barrel in vS1. (B) Widefield two-photon images of cranial window with barrel centers denoted before (left) and after (right) C2 barrel lesion. Scale bar, 0.5 mm. (C) Coronal section showing vS1 lesion in an example animal, with vS1 outline based on the Allen Brain Atlas. Scale bar, 500 μm. (D) Mean touch-evoked ΔF/F for whisker C2 protractions (left) and whisker C3 protractions (right) for all responsive neurons in an example mouse (different from B) with neurons colored by touch type in vS2 the session prior to (top) and after (bottom) a vS1 lesion targeting the whisker C3 barrel. (E) Relative change in touch-evoked ΔF/F averaged across touch types in vS2 before and after vS1 lesion. Bar, mean (overall: n = 8 mice, whisker specific: n = 7 mice). Orange, neurons that preferred to respond to touches by lesioned barrel’s preferred whisker. Gray, neurons that preferred the unlesioned barrel’s whisker. p values indicated for two-sided paired (paired within animal) t tests: **p < 0.01. (F) Same as in (E) but broken up by touch subpopulation. (G) As in (F) but for relative change in touch type fraction in vS2 before and after vS1 lesion.

Vibrissal S2 enhances touch responses in somatotopically matched regions of vS1. If vS1 similarly enhances vS2 responses, this would imply that the two areas recurrently amplify spatially specific cortical touch responses. In contrast to vS2, vS1 somatotopy is sufficiently clear to target individual barrel columns.^42^ We therefore focally lesioned a single vS1 barrel and recorded vS2 touch responses before and after this lesion (Figures 5A–5C).

We imaged a single three-plane vS2 subvolume spanning 180 μm in total depth (Table S1) in behaving mice both before and after lesion, yielding 1,244 ± 152 vS2 neurons in approximately 300- by 600-μm subregions across eight mice (Figure 5D). Lesioning a single whisker’s barrel column in vS1 reduced the relative mean touch-evoked ΔF/F in vS2 (Figure 5E; p = 0.007, n = 8 mice, t test comparing relative change to 0). When we restricted our analysis to neurons preferring the whisker of either the lesioned or spared barrel, we found that the touch-evoked ΔF/F response to the lesioned barrel’s whisker declined (Figure 5E, p = 0.002, n = 7 mice), whereas the unlesioned barrel’s whisker response did not (p = 0.376, lesioned vs. unlesioned, p = 0.009). Reduced responses were observed in all three populations of touch neurons (Figure 5F, n = 8 mice, USW, p = 0.009; BSW, p = 0.006; MW, p = 0.006, t test comparing relative change to 0). After vS1 lesion, we observed a significant decline in the fraction of USW cells (Figure 5G; p = 0.003), whereas the fraction of BSW (p = 0.227) and MW cells (p = 0.057) remained stable. Barrel-targeted vS1 lesions therefore produce a decline in aggregate vS2 touch responsiveness, with a larger effect among neurons responding to touch by the principal whisker of the targeted barrel. The vS1 to vS2 projection thus broadly influences touch-responsive neurons in vS2, with an especially pronounced impact on USW neurons in vS2.

In a subset of mice (n = 7) with vS1 lesions, we evaluated the local lesion effect by imaging tissue adjacent to the lesion site (1,610 ± 213 neurons). Though touch responses in the target barrel were eliminated, much of the touch response in the adjacent tissue remained, with a distance-dependent decline observed only for touch responses to the whisker of the targeted barrel (Figure S8). Traditional cortical lesions, which remove far larger volumes of tissue than our approach, can result in degradation of thalamocortical neurons.^43,44^ We therefore examined several markers of thalamocortical degeneration: changes in Nissl stain reactivity, along with both microglial (Iba1) and astrocytic (GFAP) immunoreactivity (STAR Methods). We did not observe any signs of thalamocortical degeneration (Figure S9). As with vS2 lesions, vS1 lesions did not alter vibrissal kinematics (Figure S4), the vS2 correlation structure (Figure S5), or the whisking-responsive population (Figure S6). We also found no change in the turnover rate of touch cell types in vS2 after vS1 lesion (Figure S7). Reduced vS2 touch responsiveness following vS1 lesions is thus most likely due to the loss of direct input from vS1.

The vS1 to vS2 projection enhances the downstream touch response across touch cell types, with barrel-specific effects demonstrating the somatotopic specificity of this projection. Therefore, vS1 and vS2 recurrently amplify touch responses across iso-somatotopic subregions of cortex.

## DISCUSSION

We studied interactions across iso-somatotopic subregions of L2/3 in mouse vS1 and vS2. We found that both vS1 and vS2 contain a sparse, mostly superficial, population of broadly tuned neurons that respond robustly to touch by both whiskers and exhibit high levels of synchrony (Figure 2). This population carries a large proportion of the interareal touch signal in both directions, especially from vS2 to vS1, and its synchrony suggests that it is especially effective at influencing downstream targets^36^ (Figure 3). Lesioning^41^ the subregion of either area that responded to touch by the spared whiskers resulted in a decline in touch response in the iso-somatotopic subregion of the other area, with whisker-specific vS1 lesions producing whisker-specific vS2 effects (Figures 4 and 5). We therefore propose that intracolumnar feedforward processing from L4 to L2 in vS1 and vS2 broadens receptive fields, yielding a sparse population of broadly tuned neurons that then recurrently amplify the touch response across iso-somatotopic subregions of both areas.

Topography-respecting excitatory projections between areas are common in cortex.^2,45^ What computations might such circuits subserve? First, topography-respecting circuitry could contribute to spatial attention.^16,17^ Signals from higher order areas augmenting location-specific responses in vS2, for instance, could propagate via such circuitry to produce enhanced responses in somatotopically matched regions of vS1 or other areas connected in this manner. Second, topography-respecting excitation could contribute to object recognition^19,20^ by elevating the sensitivity of topographically matched subregions of other cortical areas responding to the same stimulus. Because vS1 and vS2 encode distinct features of touch,^24^ iso-somatotopic recurrent circuitry ensures that both responses concurrently reach higher-order downstream areas putatively performing object recognition. Finally, such circuitry could act as a substrate to “bind”^18^ activity representing the same stimulus across disparate cortical regions, resulting in a cohesive percept.

We find that interareal projections are dominated by a sparse population of broadly tuned cells that presumably emerge due to feedforward processing from L4 to L2.^33^ These cells are likely to be locally recurrently coupled,^10^ resulting in elevated synchrony and greater downstream influence.^36^ For the vS1 to vS2 projection, BSW and MW cells together accounted for the majority of projection activity.^46^ For the vS2 to vS1 projection, MW cells constituted the outright majority of projection activity. USW cells, though most numerous in both areas, contributed marginally. It is therefore likely that broadly tuned neurons coordinate and relay the touch output of each area. Our work therefore suggests that iso-somatotopic recurrence across areas responding to specific touch stimuli is mediated by a broadly tuned population comprising 1%–2% of L2/3 neurons in a given area,^33^ though further experiments are needed to confirm that these particular cells are causally responsible for recurrent amplification across vS1 and vS2. This argues against interpretations of interareal connectivity in which feedforward projections are narrowly tuned and feedback projections are broadly tuned. This discrepancy could be due to actual differences between species, differences between sensory modalities, or it could be due to technical issues such as the failure to detect and compare topographically matched neurons across two areas to a sufficient degree, the oversampling of infragranular layers, and the failure to distinguish projecting neurons in many studies.

In visual cortex, feedback projections can suppress responses among iso-retinotopic subregions of cortex,^3,47,48^ and many interareal projections recruit inhibition in the target area.^49,50^ Moreover, iso-retinotopic axonal projections appear to follow connectivity patterns that may preferentially amplify stimuli moving in specific directions across the visual field.^51^ Some pairs of areas may thus implement specific computations based on distinct patterns of both excitatory and inhibitory connectivity. Due to the difficulty of recording precisely matched sites across areas and the frequent lack of behavioral engagement and laminar specificity, it remains unclear if the discrepancy between our results and those observed in the visual system are due to genuine circuit differences or due to differences in experimental approach.

Though L2/3-to-L2/3 connectivity is observed among adjacent areas,^12,15^ L5-to-L2/3, L2/3-to-L4, and other connections are also observed.^1,2,13,52^ Modeling work suggests that projections connecting specific layers make distinct contributions to interareal interactions.^53^ Vibrissal S1 and vS2 are also interconnected across many layers: retrograde injections across the cortical depth of one area result in varying degrees of labeling in most layers of the other area.^2^ Our retrograde injections were specifically targeted to L2/3, which resulted in most labeling being confined to L2/3 in the other area along with extensive intracolumnar labeling in other layers. This rich intracolumnar connectivity^54^ suggests that other layers indirectly contribute to the recurrent amplification revealed by our lesions. Moreover, both thalamocortical and corticothalamic projections are often topographically organized,^55–57^ providing a further putative recurrent loop between vS1 and vS2. Finally, although our lesions typically target superficial layers,^41^ dendrites of many neurons with somata below L2/3 will be cut, and L2/3 input will be eliminated, so that nearly all neurons below the lesion site are likely to exhibit reduced touch responsiveness. It is thus likely that a substantial portion of the lesion effect is due to polysynaptic pathways, either within cortex or via thalamus. Projections between specific pairs of areas and laminae^52,58^ may contribute to distinct computations, with L2/3-mediated interactions driving recurrent amplification, and other layer pairs potentially performing either complementary or distinct computations.

We find that vS1 and vS2 recurrently amplify the interareal cortical touch response and that a sparse population of broadly tuned superficial L2/3 neurons that exhibit elevated synchrony dominates the interareal transfer of touch activity. During somatosensation, topography-respecting projections thus augment the cortical response to localized touch across iso-somatotopic subregions of vS1 and vS2.

### Limitations of the study

Because we trim all but two of the approximately seven whiskers whose barrels our vS1 imaging window encompasses, some neurons that are MW are classified as single whisker, making it likely that the influence of MW cells is even more pronounced than reported here. In our retrograde injection experiments, we find that vS1 projections to a focal site in vS2 originate from many barrels, which makes it likely that multiwhisker integration is also happening through vS1 to vS2 projections. In contrast, vS2 projections to vS1 originate from a far more spatially focal source, and the discrepancy in map size is insufficient to account for this.^23^ Vibrissal S2 therefore seems to be receiving information from many whisker barrels, consistent with vS2 being a site for multiwhisker integration.^23,24^ The fact that vS2 lesions decrease touch activity in vS1 but do not eliminate MW responses suggests that both local and interareal pathways are important for multiwhisker integration.

Results from anesthetized mice where 24 whiskers were each passively stimulated revealed fast vS1 responses to whisker touch, whereas vS2 responses were more prolonged, consistent with a potential role for vS2 in multiwhisker integration.^24^ In addition, passive whisker stimulation during voltage-sensitive dye imaging in anesthetized mice revealed that activity propagates to more distant sections within a cortical area on a faster time-scale in vS2 than in vS1, likely due to the smaller size of vS2, which should facilitate multiwhisker integration.^23^ In our active whisking experiments, we find more MW neurons in vS2 than vS1. However, we also see evidence of multiwhisker integration in vS1 itself. It is possible that the receptive fields of MW neurons in vS2 are much larger than those of MW cells in vS1, but this cannot be resolved with our two-whisker task design.

Two potential sources of off-target damage may have contributed to the post-lesion response declines. First, lesions could have driven degeneration of thalamocortical afferents, which is often observed following larger somatosensory lesions.^43,44^ We did not observe thalamic degeneration following our lesions, with mild damage only observed following a far larger lesion than the ones used here (Figure S9). Second, lesions may damage axons of projecting neurons originating in the other area, potentially reducing those neurons’ responses. Were this the mechanism of response decline, we would expect the dominant projection type—MW touch neurons—to show the largest decline in response in the unlesioned areas, which contrasts with what we observe. Moreover, the lack of impact on whisking neurons (Figure S6), which also project in both directions, argues against this interpretation. Nevertheless, some portion of the observed effect may be due to unanticipated off-target effects.

## STAR★METHODS

### RESOURCE AVAILABILITY

#### Lead contact

Further information and requests for resources and reagents should be directed to the lead contact, Simon Peron (speron@nyu.edu).

#### Materials availability

This study did not generate new unique reagents.

#### Data and code availability

- Data reported in this paper will be provided upon reasonable request to the Lead Contact.
- Source code used in this paper has been deposited at http://github.com/peronlab and is publicly available as of the date of publication.
- Any additional information required to reanalyze the data reported in this work paper is available from the Lead Contact upon request.

### EXPERIMENTAL MODEL AND SUBJECT DETAILS

#### Animals

Adult Ai162 (JAX 031562) X Slc17a7-Cre (JAX X 023527)^28^ mice (18 female, 21 male) were used (Table S1). These mice express GCaMP6s exclusively in excitatory cortical neurons. Breeders were fed a diet that included doxycycline (625 mg/kg doxycycline; Teklad) so that mice received doxycycline until they were weaned, suppressing transgene expression throughout development. Animals were kept on a reverse light cycle and maintenance of animal colonies was performed by both the laboratory and veterinary staff. Any animals who were water restricted (see Behavior) were given 1 mL of water per day, with necessary adjustments made to maintain a weight of 80–90% of pre-restriction baseline. Animals of both sexes were used, though sample sizes were too small to compare results across males and females. All animal procedures and protocols were approved by New York University’s University Animal Welfare Committee.

### METHOD DETAILS

#### Surgery

Cranial window and headbar implantation occurred in mice 6–10 weeks old under isoflurane anesthesia (3% induction, 1–2% maintenance). A dental drill (Midwest Tradition, FG 1/4” drill bit) was used to make a circular craniotomy in the left hemisphere over vS1 and vS2 (3.5 mm in diameter; vS1 center: 3.5 mm lateral, 1.5 mm posterior from bregma; vS2 center: 4.7 mm lateral, 1.5 mm posterior from bregma). A triple-layer cranial window (4.5 mm external diameter, 3.5 mm inner diameter, #1.5 coverslip; two smaller windows were adhered to each other and the larger window with Norland 61 UV glue) was positioned over the craniotomy. The headbar and window were both affixed to the skull using dental acrylic (Orthojet, Lang Dental). Mice were post-operatively injected with 1 mg/kg of buprenorphine SR and 5 mg/kg of ketoprofen.

#### Retrograde labeling

Retrograde viral injections were performed in untrained mice who had previously been implanted with a cranial window and had been trimmed to two whiskers (C2, C3). After surgical recovery, mice were run for one session on the imaging rig to identify either the touch patch in vS2 or the C2 and C3 barrels in vS1 (see Area Identification). For vS1 injections, we targeted the center of one of the two barrels (C2 or C3). For vS2 injections, we targeted the center of the patch of vS2 responsive to C2 and C3 touch. In both cases, the window was drilled off and a durotomy was performed. We injected 100 nL of rAAV2-*retro*-FLEX-tdTomato (pAAV-FLEX-tdTomato was a gift from Edward Boyden, Addgene viral prep # 28306-AAVrg, 1×10^13^ vg/mL diluted 1:50 in 1xPBS; http://n2t.net/addgene:28306; RRID:Addgene_28306) into the target area at a depth of 200 μm and a rate of 20 nL/min (Narishige MO-10 hydraulic micromanipulator). Injection was performed using a glass capillary pulled with a micropipette puller (P-97, Sutter) and beveled to a tip with a ~25° angle and 25 μm diameter. This was backfilled with mineral oil and 2 μL of the virus was pulled into the tip. The pipette was lowered into the target area at a rate of 300 μm/min which was followed by a 1-min delay before injection began. An identical, new, triple layer cranial window was placed over the craniotomy as before and re-affixed to the skull with dental acrylic. A subset of retrogradely injected animals were perfused (see “Histology and immunohistochemistry” in STAR Methods) and imaged on a confocal microscope (model SP5, Leica) using a 20× objective (Figure S3).

#### Behavior

After surgical recovery, mice were water restricted and placed on a reverse light cycle. They were typically given 1 mL of water per day with small adjustments made to keep weight at 80–90% of pre-restriction baseline. Mice that had not been previously trimmed were trimmed to whiskers C2 and C3 and subsequently trimmed every 2–3 days.

Water-restricted mice were habituated to the behavioral apparatus for 2 days by head fixing them for 15–30 min and giving them free water. Mice were then trained on a two-whisker active touch task in which a pole was presented within range of one of two whiskers on every trial (Figure 1A). Pole positions both in front of and behind each whisker’s natural resting position were used to encourage both retraction and protraction touches. The pole position on each trial was randomized but approximately half of trials in a given session targeted whisker C2 and the other half targeted whisker C3. Positions in front of and behind the whisker were used with as equal a frequency as possible. Pole positions were occasionally adjusted if animals changed their resting whisker position. For the longer whisker, pole placement was beyond the reach of the shorter whisker. Though the longer whisker did occasionally touch on trials meant for the shorter whisker, we obtained large numbers of isolated touch trials for all whiskers and touch directions (Figure 1D). The pole was presented to the animal for 1 s, followed by a 2 s delay, and then a response cue (3.4 kHz, 50 ms) signaled the start of a 1 s response period. Mice would lick during the response period and would receive water randomly on ~70% of trials. This was done to increase the number of trials during which the animal was engaged. A loud (60–70 dB) white noise sound was played for 50 ms following the onset of pole movement, which encouraged appropriately timed whisking. The lickport was moved along the anterior-posterior axis via a motor (Zaber) so that it was only accessible during the response period. Naive mice whisked naturally at the onset of pole movement, likely due to the white noise sound, and would encounter the pole by chance. Over the course of a session, mice began to whisk vigorously in a stereotypical manner and subsequently lick after encountering the pole with the whisker.

A BPod state machine (Sanworks) and custom MATLAB software (MathWorks) running on a behavioral computer (System 76) controlled the task. Sounds were produced and controlled by an audio microcontroller (Bela). Three motorized actuators (Zaber) and an Arduino controlled lickport motion. Licks were detected via a custom detection circuit (Janelia).

#### Whisker videography

Whisker video was acquired with custom MATLAB software using a CMOS camera (Ace-Python 500, Basler) with a telecentric lens (TitanTL, Edmund Optics) running at 400 Hz with 640 × 352 pixel frames. The video was illuminated by a pulsed 940 nm LED (SL162, Advanced Illumination) synchronized with the camera (typical exposure and illumination duration: 200 μs). 7–9 s of each trial were recorded, which included 1s prior to pole movement, the pole in-reach period, and several seconds following pole withdrawal. Data was processed on NYU’s High Performance Computing (HPC) cluster. Whiskers were detected using the Janelia Whisker Tracker.^31^ Whisker identity assignment was then refined and evaluated using custom MATLAB software.^10,26^ Whisker curvature (κ) and angle (θ) were then calculated at specific locations along the whisker’s length. Change in curvature, Δκ, was measured relative to a resting baseline curvature which was calculated at each angle independently. This value was obtained during periods when the pole was out of reach. Automatic touch detection was then performed, and touch assignment was curated manually using a custom MATLAB user interface.^26^ Protractions were assigned negative Δκ values.

#### Two-photon imaging

A custom MIMMS two-photon microscope (Janelia Research Campus) with a 16× objective (Nikon) was used for cellular-resolution imaging. An 80 MHz titanium-sapphire femtosecond laser (Chameleon Ultra 2; Coherent) tuned to 940 nm was used, with powers out of the objective rarely exceeding 50 mW. The microscope included a Pockels cell (350-80-02, Conoptics), two galvanometer scanners (6SD11268, Cambridge Technology), a resonant scanner (6SC08KA040-02Y, Cambridge Technology), a 16× objective (N16XLWD-PF, Nikon), an emission filter for green fluorescence (FF01-510/84-30, Semrock), an emission filter for red fluorescence (FF01-650/60, Semrock), two GaAsP PMTs (H10770PB-40, Hamamatsu) and two PMT shutters (VS.14S1T1, Vincent Associates). A piezo (P-725KHDS; Physik Instrumente) was used for axial movement. Three imaging planes spanning 700-by-700 μm (512-by-512 pixels) and spaced at differential depths apart depending on the experiment were collected simultaneously at ~7 Hz; we refer to this group of planes as a ‘subvolume’. Scanimage (version 2017; Vidrio Technologies) was used to collect all imaging data, and power was depth-adjusted in software using an exponential length constant of 250 μm. Up to 4 subvolumes were imaged per animal, and each subvolume was imaged for about 100 trials before moving to the next subvolume. All subvolumes were imaged on any given imaging day. After the first day of imaging, a motion-corrected mean image was created for each plane, which was then used as the reference image for any potential following imaging days. For animals where both vS1 and vS2 were imaged, two subvolumes were employed in each area spaced 60 μm apart, for a total span of 360 μm. For projection experiments, two subvolumes were collected in the non-injected area spaced 30–40 μm apart, for a total span of 180–240 μm. For lesion experiments, one subvolume was collected in each area spaced 60 μm apart, for a total span of 180 μm (Table S1).

After acquisition, imaging data was processed on the NYU HPC cluster. First, image registration was completed for motion correction using a line-by-line registration algorithm.^26^ Segmentation was performed on one session: neurons from the first day of imaging were detected using an automated algorithm based on template convolution that identified neuron centers, after which a neuron pixel assignment algorithm that detects annular ridges given a potential neuron center^27^ was used to identify the precise edges of the neuron. All pixels, including the nucleus, were used. This initial segmentation was manually curated, establishing a reference segmentation for each plane. On subsequent imaging days, the segmentation was algorithmically transferred to the new mean images for a given plane for that day.^59^ After segmentation, ΔF/F computation and neuropil subtraction were performed. The neuropil-corrected ΔF/F trace was used for subsequent analyses.

In animals with projection labeling, neurons were classified as projecting if their mean tdTomato fluorescence exceeded a manually selected threshold. For each pixel on a plane, the cross-session mean tdTomato fluorescence was calculated. For any given neuron, its ‘redness‘ was taken as the mean red fluorescence value for its constituent pixels in this mean image. A manual user interface that flagged neurons exceeding a threshold red fluorescence was used to find the threshold which appropriately partitioned tdTomato expressing neurons from non-expressing neurons in each animal.

#### Lesions

A 1040 nm 80 MHz fs laser (Fidelity HP, Coherent) was focused at a depth of 200–300 μm for 10–20 s at 1–1.5 W power (out of objective) to produce lesions. Vibrissal S1 lesions were made by centering the laser on a target barrel; vS2 lesions were made by centering the laser on the most responsive touch patch in the vS2 field of view. Sham lesions were performed in visual areas medial and posterior to vS1. Lesions were performed in awake, head fixed, durotomized animals sitting in the behavioral apparatus. Animals were monitored for signs of distress or discomfort. Typically, the lesion was performed at the end of a behavioral/imaging session. Post-lesion measurements were taken during the next session, approximately 24 h after lesion. Previous studies in the lab have shown that there is an acute response near the lesion site for 1–2 h after lesioning. We have chosen the 24-h time-line to be safely away from this 2 h period and so that we can see acute changes in the brain before it has time to rewire or for plasticity to take place. This approach consistently yields lesions with a volume of 0.1–0.2 mm^3^, and when performed in animals expressing GCaMP6s, the radius of the post-lesion calcium response can be used to infer lesion extent.^41^ In animals where intentionally large lesions were made (Figure S9), we made five or more additional lesions surrounding the initial lesion using the same depth, power, and timing parameters.

#### Histology & immunohistochemistry

After several days of imaging, some animals were perfused with paraformaldehyde (4% in PBS) and postfixed overnight. A vibratome (Leica) was used to cut coronal sections 100-mm thick which were mounted on glass slides with Vectashield antifade mounting media containing DAPI (Vector Laboratories). These sections were imaged on a fluorescent light microscope (VS120, Olympus). Slices were used to determine lesion location or injection spread. To ensure proper areal identification, all images were registered to the Allen Mouse Brain Common Coordinate framework using the SHARP-track pipeline.^60^

For Figure S9, two mice used in this study were perfused 72 h after vS1 lesion (after all imaging was complete). Two additional mice were given exorbitantly large vS1 lesions (see: Lesions) and perfused 72 h after lesion. A vibratome (Leica) was used to cut 50-μm thick sections and sections that included the lesion were either used for immunohistochemistry or Nissl staining.

For immunohistochemistry, slices were incubated overnight under agitation with primary antibody that was made in 1% bovine serum albumin and 0.05% sodium azide. Slices were labeled with either rabbit anti-Iba1 (Wako; 019–19741) or mouse anti-GFAP (glial fibrillary acidic protein; Sigma G3893)^61^ antibodies. After incubation, slices were washed and incubated in secondary antibody (1:500) conjugated to Alexa Fluor 647. Finally, slices were rinsed and mounted using an antifade mounting media (Vector Laboratories), and subsequently imaged using an Olympus VS120 microscope and a Leica SP5 confocal microscope. Primary antibodies: rabbit anti-Iba1 (019–19741; Wako; 1:500 dilution), mouse monoclonal anti-GFAP (G3893; Sigma-Aldrich; 1:1,000 dilution). Secondary antibodies: goat anti-Rabbit, Alexa Fluor 647 (Thermo Fisher A-21244), and goat anti-Mouse, Alexa Fluor 647 (Thermo Fisher A-21235).

For Nissl staining, slices were immediately mounted and then deparaffinized with xylene, and sequentially rinsed in varying concentrations of ethanol and distilled water. Sections were then Nissl stained by submersion of slides in 0.125% cresyl violet, followed by further rinsing. Dried and stained slides were imaged on the Olympus VS120 microscope in bright-field mode.

#### Area identification

The locations of vS1 (including individual barrel locations) and vS2 were identified by measuring the GCaMP6s ΔF/F at coarse resolution (4× objective, Nikon; field of view, 2.2×2.2 mm) on the two-photon microscope while the whiskers were deflected individually with a pole. Imaging was performed for a single imaging plane at 28 Hz. This was done in awake mice not engaged in any task. For injections and lesions, we then briefly imaged at cellular resolution using volumetric imaging to further restrict ourselves to the relevant injection or lesion target. Whiskers were individually deflected with the pole for approximately 100–200 trials while we imaged in one subvolume with three planes and 60 μm spacing. We then analyzed the touch response in individual neurons (see “Touch and whisking responsiveness” in STAR Methods), identifying a single barrel for vS1 injections or lesions, or the vS2 touch patch.

Vibrissal S1 and vS2 are similarly extensive along the anterior-posterior (AP) axis, but vS2 is relatively compressed along the mediolateral (ML) axis.^2,23^ Within our imaging fields of view, we therefore selected a 600-by-600 μm field of view for vS1 centered on the C2/C3 border, and a 600 μm (AP axis) by 300 mm (ML axis) field of view for vS2. This ensured that we were not analyzing vS1 cells in our vS2 field of view and that we did not underestimate vS2 cell fractions. This restricted area overlapped with the area in vS2 expressing retrogradely labeled neurons following vS1 injections.

#### Touch and whisking responsiveness

A neuron was classified as responsive or non-responsive for a particular touch trial by comparing ΔF/F_baseline_, the mean ΔF/F for the 6 frames (0.85 s) preceding the first touch, to ΔF/F_post-touch_, the mean ΔF/F for the period between the first touch and two frames after the final touch (interframe interval, ~143 ms). For each neuron, we computed the standard deviation of ΔF/F across all pre-touch frames (6 frames prior to first touch), yielding a noise estimate, σ_baseline_. A neuron was considered responsive on a trial if the ΔF/F_post-touch_ exceeded ΔF/F_baseline_ for that trial by at least 2*σ_baseline_. Neurons that were responsive on at least 5% of trials for a given touch type were considered part of the responsive pool for that type.

This 5% threshold was chosen to match the touch cell type distribution previously observed in vS1 using an encoding model.^33^ In this work, we adopted a response probability threshold because of its relative simplicity. Increasing the threshold to 10% or 20% increases the fraction of multiwhisker neurons as unidirectional single whisker neurons have a lower response level, whereas reducing the threshold has the opposite effect.

To compute a neuron’s mean touch-evoked ΔF/F, we incorporated the response to all touch types for which that neuron was significantly responsive. In all cases, touch-evoked ΔF/F was measured as ΔF/F_post-touch_-ΔF/F_baseline_ for a given trial. For a given touch type (C2P, C2R, C3P, and C3R), mean touch-evoked ΔF/F was calculated using trials where only that touch type occurred. For unidirectional single whisker cells, we used the mean touch-evoked ΔF/F for the single touch type it responded to. For bidirectional single whisker cells, we used the mean touch-evoked ΔF/F averaged across the two directions for the whisker the cell responded to, weighing both touch types equally. Finally, for multiwhisker cells, we first calculated the mean touch-evoked ΔF/F for all the touch types to which that cell responded, then took the mean across these values.

Whisking responsive neurons (Figure S6) were classified in the same way as described for touch responsive cells, with the key difference of aligning responses based on whisking bout onset as opposed to touch. Whisking bouts were defined as periods where the amplitude of whisking derived from the Hilbert transform^62^ exceeded 5°. As before, ΔF/F_baseline_ and ΔF/F_post-whisking-onset_ were compared and a cell was considered whisking responsive overall if it was responsive on at least 5% of trials with whisking.

#### Correlation analysis

Pearson correlations were calculated across neuron pairs either around the time of touch or restricted to time points outside of touch (‘spontaneous’ epoch). In all cases, correlations were computed using the ΔF/F values at all included timepoints. Periods outside of touch were defined as any timepoints that did not fall between 1 s before and 10 s after a touch. For touch correlations, a mean correlation was calculated using a window that began 1 s prior to first touch onset and ending 4 s after final touch offset. Four correlations were computed per neuron, one each for C2P, C2R, C3P, and C3R touches. For unidirectional single whisker neurons, we only used the value for the touch type the neuron responded to. For bidirectional single whisker and multiwhisker neurons, we took the grand mean of the mean correlations from trials with isolated touches for the touch types the neuron was considered responsive to.

Because only individual subvolumes were recorded simultaneously, we aggregated across subvolumes in some cases. For non-projection analyses, we only employed the superficial subvolume. For projections, we imaged superficial L2/3 with two subvolumes due to the relatively low yield of retrograde labeling, and so we aggregated these when possible within animals by using the grand mean of individual population correlations across subvolumes. We applied a 5-neuron minimum within a given category for correlation computation. This did not impact most analyses, but for projection-based correlations, it precluded the analysis of bidirectional single whisker neurons in vS2 altogether and did result in the exclusion of some subvolumes. If only one subvolume met this criterion for an animal, that subvolume was used for the correlation values for that animal. If both subvolumes met the criteria, correlations for that animal were computed as the mean across the two subvolumes. If both subvolumes failed to meet the cell count criteria, the animal was excluded from analysis.

#### Analysis of the fraction of response carried by each population

For the analyses in Figures 2G, 2H, and 3G, for each animal, we found the response of each touch neuron (Figure 2) or each projecting neuron (Figure 3) to each type of touch that neuron responded to for every touch trial. For each trial, we took the mean response to touch for each type of touch that the neuron responded to, and that mean was the mean response to touch for that neuron on a given trial. For each neuron we took the mean across trials, and then sorted neurons based on their touch response classification (USW, BSW, or MW). By summing the mean response of each neuron in each group we found the overall mean response to touch in each touch type category. We took the sum of the mean response of all touch or projecting neurons to be the total touch response of the touch or projecting neuron populations. The division of the mean response to touch in each category with the total response in an area gives the fraction of response carried by each cell type in that area.

### QUANTIFICATION AND STATISTICAL ANALYSIS

We used the two-tailed paired t test for comparisons across matched groups, where pairing was typically within-animal. The two-sample t test was used to compare between distinct samples. In a few cases, we used a t test against 0 or 1 to compare population change to chance. All statistical analysis was performed using MATLAB, and a list of exactly which animals are used in each experiment can be found in Table S1. Statistical tests are described in the results section and in the legends of relevant figures. Parametric statistics were used throughout; no tests were used to establish normality.

## Supplementary Material

1

## Figures and Tables

**Table:** KEY RESOURCES TABLE

REAGENT or RESOURCE	SOURCE	IDENTIFIER
Antibodies		
rabbit anti-Iba1	Wako	Cat# 019–19741; RRID: AB_839504
mouse monoclonal anti-GFAP	Sigma-Aldrich	Cat# G3893; RRID: AB_477010
goat anti-Rabbit	Thermo Fisher	Cat# A-21244; RRID:AB_2535812
goat anti-Mouse	Thermo Fisher	Cat# A-21235; RRID:AB_2535804
Bacterial and virus strains		
pAAV-FLEX-tdTomato	Addgene	28306-AAVrg
Chemicals, peptides, and recombinant proteins		
Doxycycline (625 mg/kg)	Teklad	TD.01306
Buprenorphine SR	Zoo Pharm	N/A
Ketoprofen (50 mL, 100 mg/mL)	Henry Schein	10004031
Experimental models: Organisms/strains		
Transgenic mouse, Ai162	Jackson Labs	JAX 031562
Transgenic mouse, Slc17a7-Cre	Jackson Labs	JAX 023527
Software and algorithms		
Scanimage	Vidrio	Version: 2017
MATLAB	MathWorks	Versions: 2015, 2017
Source code for figures	This manuscript	Zenodo https://doi.org/10.5281/zenodo.10034649
SHARP-track pipeline	https://www.biorxiv.org/content/10.1101/447995v1	N/A
Janelia whisker tracker	Janelia	N/A
Other		
One-axis oil hydraulic micromanipulator	Narishige	MO-10
Glass capillary	Drummond Scientific	5-000-2010
Micropipette puller	Sutter	P-97
Confocal microscope	Leica	SP5
BPod state machine	Sanworks	0.7
Behavioral computer	System 76	Wild Dog Pro
Audio microcontroller	Bela	Bela Starter Kit
Motorized actuators	Zaber	NA11B60-T4-MC04
Microcontroller	Arduino	Uno; Due
Custom lick detection circuit	Janelia Research Campus	N/A
CMOS camera	Basler	Ace-Python 500
Telecentric lens	Edmund Optics	TitanTL
MIMMS two-photon microscope	Janelia Research Campus	N/A
Femtosecond laser	Coherent	Chameleon Ultra 2

## Highlights

- Vibrissal S1 and S2 contain sparse populations of broadly tuned touch neurons
- Broadly tuned neurons carry a large fraction of each area’s touch response
- Broadly tuned touch neurons carry most of the touch activity between vS1 and vS2
- Topographically matched regions of vS1 and vS2 amplify one another’s touch responses

## References

[1] Felleman DJ, and Van Essen DC (1991). Distributed hierarchical processing in the primate cerebral cortex. Cereb. Cortex 1, 1–47. 1822724 10.1093/cercor/1.1.1-a

[2] Minamisawa G, Kwon SE, Chevee M, Brown SP, and O’Connor DH (2018). A Non-canonical Feedback Circuit for Rapid Interactions between Somatosensory Cortices. Cell Rep. 23, 2718–2731.e2716. 29847801 10.1016/j.celrep.2018.04.115 PMC6004823

[3] Klink PC, Dagnino B, Gariel-Mathis MA, and Roelfsema PR (2017). Distinct Feedforward and Feedback Effects of Microstimulation in Visual Cortex Reveal Neural Mechanisms of Texture Segregation. Neuron 95, 209–220.e3. 28625487 10.1016/j.neuron.2017.05.033

[4] Mountcastle VB (1997). The columnar organization of the neocortex. Brain 120, 701–722. 9153131 10.1093/brain/120.4.701

[5] Yu J, Hu H, Agmon A, and Svoboda K (2019). Recruitment of GABAergic Interneurons in the Barrel Cortex during Active Tactile Behavior. Neuron 104, 412–427.e4. 31466734 10.1016/j.neuron.2019.07.027 PMC6813892

[6] Pouchelon G, Gambino F, Bellone C, Telley L, Vitali I, Lüscher C, Holtmaat A, and Jabaudon D (2014). Modality-specific thalamocortical inputs instruct the identity of postsynaptic L4 neurons. Nature 511, 471–474. 24828045 10.1038/nature13390

[7] O’Connor DH, Peron SP, Huber D, and Svoboda K (2010). Neural activity in barrel cortex underlying vibrissa-based object localization in mice. Neuron 67, 1048–1061. 20869600 10.1016/j.neuron.2010.08.026

[8] Ko H, Hofer SB, Pichler B, Buchanan KA, Sjöström PJ, and Mrsic-Flogel TD (2011). Functional specificity of local synaptic connections in neocortical networks. Nature 473, 87–91. 21478872 10.1038/nature09880 PMC3089591

[9] Marshel JH, Kim YS, Machado TA, Quirin S, Benson B, Kadmon J, Raja C, Chibukhchyan A, Ramakrishnan C, Inoue M, (2019). Cortical layer-specific critical dynamics triggering perception. Science 365, eaaw5202. 31320556 10.1126/science.aaw5202 PMC6711485

[10] Peron S, Pancholi R, Voelcker B, Wittenbach JD, Ólafsdóttir HF, Freeman J, and Svoboda K (2020). Recurrent interactions in local cortical circuits. Nature 579, 256–259. 32132709 10.1038/s41586-020-2062-x PMC8092186

[11] Douglas RJ, and Martin KAC (2007). Recurrent neuronal circuits in the neocortex. Curr. Biol 17, R496–R500. 17610826 10.1016/j.cub.2007.04.024

[12] Yamashita T, Vavladeli A, Pala A, Galan K, Crochet S, Petersen SSA, and Petersen CCH (2018). Diverse Long-Range Axonal Projections of Excitatory Layer 2/3 Neurons in Mouse Barrel Cortex. Front. Neuroanat 12, 33. 29765308 10.3389/fnana.2018.00033 PMC5938399

[13] Winnubst J, Bas E, Ferreira TA, Wu Z, Economo MN, Edson P, Arthur BJ, Bruns C, Rokicki K, Schauder D, (2019). Reconstruction of 1,000 Projection Neurons Reveals New Cell Types and Organization of Long-Range Connectivity in the Mouse Brain. Cell 179, 268–281.e13. 31495573 10.1016/j.cell.2019.07.042 PMC6754285

[14] Yamashita T, Pala A, Pedrido L, Kremer Y, Welker E, and Petersen CCH (2013). Membrane potential dynamics of neocortical projection neurons driving target-specific signals. Neuron 80, 1477–1490. 24360548 10.1016/j.neuron.2013.10.059

[15] Markov NT, Vezoli J, Chameau P, Falchier A, Quilodran R, Huissoud C, Lamy C, Misery P, Giroud P, Ullman S, (2014). Anatomy of hierarchy: feedforward and feedback pathways in macaque visual cortex. J. Comp. Neurol 522, 225–259. 23983048 10.1002/cne.23458 PMC4255240

[16] Moore T, and Armstrong KM (2003). Selective gating of visual signals by microstimulation of frontal cortex. Nature 421, 370–373. 12540901 10.1038/nature01341

[17] Boshra R, and Kastner S (2022). Attention control in the primate brain. Curr. Opin. Neurobiol 76, 102605. 35850060 10.1016/j.conb.2022.102605 PMC13014281

[18] Robertson LC (2003). Binding, spatial attention and perceptual awareness. Nat. Rev. Neurosci 4, 93–102. 12563280 10.1038/nrn1030 PMC3373472

[19] Roelfsema PR (2006). Cortical algorithms for perceptual grouping. Annu. Rev. Neurosci 29, 203–227. 16776584 10.1146/annurev.neuro.29.051605.112939

[20] Mumford D (1992). On the computational architecture of the neocortex. II. The role of cortico-cortical loops. Biol. Cybern 66, 241–251. 1540675 10.1007/BF00198477

[21] Woolsey TA, and Van der Loos H (1970). The structural organization of layer IV in the somatosensory region (SI) of mouse cerebral cortex. The description of a cortical field composed of discrete cytoarchitectonic units. Brain Res. 17, 205–242. 4904874 10.1016/0006-8993(70)90079-x

[22] Kwon SE, Yang H, Minamisawa G, and O’Connor DH (2016). Sensory and decision-related activity propagate in a cortical feedback loop during touch perception. Nat. Neurosci 19, 1243–1249. 27437910 10.1038/nn.4356 PMC5003632

[23] Hubatz S, Hucher G, Shulz DE, and Férézou I (2020). Spatiotemporal properties of whisker-evoked tactile responses in the mouse secondary somatosensory cortex. Sci. Rep 10, 763. 31964984 10.1038/s41598-020-57684-6 PMC6972923

[24] Goldin MA, Harrell ER, Estebanez L, and Shulz DE (2018). Rich spatio-temporal stimulus dynamics unveil sensory specialization in cortical area S2. Nat. Commun 9, 4053. 30282992 10.1038/s41467-018-06585-4 PMC6170455

[25] Chen JL, Carta S, Soldado-Magraner J, Schneider BL, and Helmchen F (2013). Behaviour-dependent recruitment of long-range projection neurons in somatosensory cortex. Nature 499, 336–340. 23792559 10.1038/nature12236

[26] Peron SP, Freeman J, Iyer V, Guo C, and Svoboda K (2015). A Cellular Resolution Map of Barrel Cortex Activity during Tactile Behavior. Neuron 86, 783–799. 25913859 10.1016/j.neuron.2015.03.027

[27] Chen TW, Wardill TJ, Sun Y, Pulver SR, Renninger SL, Baohan A, Schreiter ER, Kerr RA, Orger MB, Jayaraman V, (2013). Ultrasensitive fluorescent proteins for imaging neuronal activity. Nature 499, 295–300. 23868258 10.1038/nature12354 PMC3777791

[28] Daigle TL, Madisen L, Hage TA, Valley MT, Knoblich U, Larsen RS, Takeno MM, Huang L, Gu H, Larsen R, (2018). A Suite of Transgenic Driver and Reporter Mouse Lines with Enhanced Brain-Cell-Type Targeting and Functionality. Cell 174, 465–480.e22. 30007418 10.1016/j.cell.2018.06.035 PMC6086366

[29] Petersen CCH (2007). The functional organization of the barrel cortex. Neuron 56, 339–355. 17964250 10.1016/j.neuron.2007.09.017

[30] Kuchibhotla KV, Gill JV, Lindsay GW, Papadoyannis ES, Field RE, Sten TAH, Miller KD, and Froemke RC (2017). Parallel processing by cortical inhibition enables context-dependent behavior. Nat. Neurosci 20, 62–71. 27798631 10.1038/nn.4436 PMC5191967

[31] Clack NG, O’Connor DH, Huber D, Petreanu L, Hires A, Peron S, Svoboda K, and Myers EW (2012). Automated tracking of whiskers in videos of head fixed rodents. PLoS Comput. Biol 8, e1002591. 22792058 10.1371/journal.pcbi.1002591 PMC3390361

[32] Severson KS, Xu D, Van de Loo M, Bai L, Ginty DD, and O’Connor DH (2017). Active Touch and Self-Motion Encoding by Merkel Cell-Associated Afferents. Neuron 94, 666–676.e9. 28434802 10.1016/j.neuron.2017.03.045 PMC5528144

[33] Voelcker B, Pancholi R, and Peron S (2022). Transformation of primary sensory cortical representations from layer 4 to layer 2. Nat. Commun 13, 5484. 36123376 10.1038/s41467-022-33249-1 PMC9485231

[34] Clancy KB, Schnepel P, Rao AT, and Feldman DE (2015). Structure of a single whisker representation in layer 2 of mouse somatosensory cortex. J. Neurosci 35, 3946–3958. 25740523 10.1523/JNEUROSCI.3887-14.2015 PMC4348190

[35] Lefort S, Tomm C, Floyd Sarria JC, and Petersen CCH (2009). The excitatory neuronal network of the C2 barrel column in mouse primary somatosensory cortex. Neuron 61, 301–316. 19186171 10.1016/j.neuron.2008.12.020

[36] Zandvakili A, and Kohn A (2015). Coordinated Neuronal Activity Enhances Corticocortical Communication. Neuron 87, 827–839. 26291164 10.1016/j.neuron.2015.07.026 PMC4545497

[37] Aronoff R, Matyas F, Mateo C, Ciron C, Schneider B, and Petersen CCH (2010). Long-range connectivity of mouse primary somatosensory barrel cortex. Eur. J. Neurosci 31, 2221–2233. 20550566 10.1111/j.1460-9568.2010.07264.x

[38] Condylis C, Lowet E, Ni J, Bistrong K, Ouellette T, Josephs N, and Chen JL (2020). Context-Dependent Sensory Processing across Primary and Secondary Somatosensory Cortex. Neuron 106, 515–525.e5. 32164873 10.1016/j.neuron.2020.02.004 PMC7210055

[39] Harrell ER, Goldin MA, Bathellier B, and Shulz DE (2020). An elaborate sweep-stick code in rat barrel cortex. Sci. Adv 6, eabb7189. 32938665 10.1126/sciadv.abb7189 PMC7494352

[40] Tervo DGR, Hwang BY, Viswanathan S, Gaj T, Lavzin M, Ritola KD, Lindo S, Michael S, Kuleshova E, Ojala D, (2016). A Designer AAV Variant Permits Efficient Retrograde Access to Projection Neurons. Neuron 92, 372–382. 27720486 10.1016/j.neuron.2016.09.021 PMC5872824

[41] Ryan L, Laughton M, Sun-Yan A, Costello S, Pancholi R, and Peron S (2022). Columnar Lesions in Barrel Cortex Persistently Degrade Object Location Discrimination Performance. eNeuro 9. ENEURO.0393, 22.2022. 10.1523/ENEURO.0393-22.2022 PMC9665881 36316120

[42] Ferezou I, Haiss F, Gentet LJ, Aronoff R, Weber B, and Petersen CCH (2007). Spatiotemporal dynamics of cortical sensorimotor integration in behaving mice. Neuron 56, 907–923. 18054865 10.1016/j.neuron.2007.10.007

[43] Cao Z, Harvey SS, Bliss TM, Cheng MY, and Steinberg GK (2020). Inflammatory Responses in the Secondary Thalamic Injury After Cortical Ischemic Stroke. Front. Neurol 11, 236. 32318016 10.3389/fneur.2020.00236 PMC7154072

[44] Necula D, Cho FS, He A, and Paz JT (2022). Secondary thalamic neuroinflammation after focal cortical stroke and traumatic injury mirrors corticothalamic functional connectivity. J. Comp. Neurol 530, 998–1019. 34633669 10.1002/cne.25259 PMC8957545

[45] Wang Q, and Burkhalter A (2007). Area map of mouse visual cortex. J. Comp. Neurol 502, 339–357. 17366604 10.1002/cne.21286

[46] Sato TR, and Svoboda K (2010). The functional properties of barrel cortex neurons projecting to the primary motor cortex. J. Neurosci 30, 4256–4260. 20335461 10.1523/JNEUROSCI.3774-09.2010 PMC6634518

[47] Javadzadeh M, and Hofer SB (2022). Dynamic causal communication channels between neocortical areas. Neuron 110, 2470–2483.e7. 35690063 10.1016/j.neuron.2022.05.011 PMC9616801

[48] Fisek M, Herrmann D, Egea-Weiss A, Cloves M, Bauer L, Lee TY, Russell LE, and Hausser M (2023). Cortico-cortical feedback engages active dendrites in visual cortex. Nature 617, 769–776. 37138089 10.1038/s41586-023-06007-6 PMC10244179

[49] D’Souza RD, Meier AM, Bista P, Wang Q, and Burkhalter A (2016). Recruitment of inhibition and excitation across mouse visual cortex depends on the hierarchy of interconnecting areas. Elife 5, e19332. 27669144 10.7554/eLife.19332 PMC5074802

[50] Naskar S, Qi J, Pereira F, Gerfen CR, and Lee S (2021). Cell-type-specific recruitment of GABAergic interneurons in the primary somatosensory cortex by long-range inputs. Cell Rep. 34, 108774. 33626343 10.1016/j.celrep.2021.108774 PMC7995594

[51] Marques T, Nguyen J, Fioreze G, and Petreanu L (2018). The functional organization of cortical feedback inputs to primary visual cortex. Nat. Neurosci 21, 757–764. 29662217 10.1038/s41593-018-0135-z

[52] Young H, Belbut B, Baeta M, and Petreanu L (2021). Laminar-specific cortico-cortical loops in mouse visual cortex. Elife 10, e59551. 33522479 10.7554/eLife.59551 PMC7877907

[53] Joglekar MR, Mejias JF, Yang GR, and Wang XJ (2018). Inter-areal Balanced Amplification Enhances Signal Propagation in a Large-Scale Circuit Model of the Primate Cortex. Neuron 98, 222–234.e8. 29576389 10.1016/j.neuron.2018.02.031

[54] Hooks BM, Hires SA, Zhang YX, Huber D, Petreanu L, Svoboda K, and Shepherd GMG (2011). Laminar analysis of excitatory local circuits in vibrissal motor and sensory cortical areas. PLoS Biol. 9, e1000572. 21245906 10.1371/journal.pbio.1000572 PMC3014926

[55] Sumser A, Mease RA, Sakmann B, and Groh A (2017). Organization and somatotopy of corticothalamic projections from L5B in mouse barrel cortex. Proc. Natl. Acad. Sci. USA 114, 8853–8858. 28774955 10.1073/pnas.1704302114 PMC5565434

[56] Agmon A, Yang LT, Jones EG, and O’Dowd DK (1995). Topological precision in the thalamic projection to neonatal mouse barrel cortex. J. Neurosci 15, 549–561. 7823163 10.1523/JNEUROSCI.15-01-00549.1995 PMC6578331

[57] Deschênes M, Veinante P, and Zhang ZW (1998). The organization of corticothalamic projections: reciprocity versus parity. Brain Res. Brain Res. Rev 28, 286–308. 9858751 10.1016/s0165-0173(98)00017-4

[58] Siu C, Balsor J, Merlin S, Federer F, and Angelucci A (2021).A direct interareal feedback-to-feedforward circuit in primate visual cortex. Nat. Commun 12, 4911. 34389710 10.1038/s41467-021-24928-6 PMC8363744

[59] Huber D, Gutnisky DA, Peron S, O’Connor DH, Wiegert JS, Tian L, Oertner TG, Looger LL, and Svoboda K (2012). Multiple dynamic representations in the motor cortex during sensorimotor learning. Nature 484, 473–478. 22538608 10.1038/nature11039 PMC4601999

[60] Shamash P, Carandini M, Harris K, and Steinmetz N (2018).A tool for analyzing electrode tracks from slice histology. Preprint at bioRxiv.

[61] Podgorski K, and Ranganathan G (2016). Brain heating induced by near infrared lasers during multi-photon microscopy. Preprint at bioRxiv. 10.1152/jn.00275.2016 PMC5009202 27281749

[62] Hill DN, Curtis JC, Moore JD, and Kleinfeld D (2011). Primary motor cortex reports efferent control of vibrissa motion on multiple timescales. Neuron 72, 344–356. 22017992 10.1016/j.neuron.2011.09.020 PMC3717360

