# Supplementary material for: Cortical circuitry mediating interareal touch signal amplification
Source: Cell Rep. Author manuscript; Available in PMC 2024 Feb 5. (PMC10842872; doi:10.1016/j.celrep.2023.113532)
Supplement: 1 [file NIHMS1954787-supplement-1.pdf]

Cell Reports, Volume *42*

Supplemental information

Cortical circuitry mediating interareal touch signal amplification

Lauren Ryan, Andrew Sun-Yan, Maya Laughton, and Simon Peron

SUPPLEMENTAL INFORMATION

Mouse	Sex	Age (wk)	Areas imaged	Imaging depth span	Neuron count	Experiments
14351	F	12	vS1/vS2	360 µm (2 sv.)	vS1: 4,416<br>vS2: 3,567	vS1 vs. vS2, vS1 lesion
14359	F	16	vS1/vS2	360 µm (2 sv.)	vS1: 4,578<br>vS2: 3,509	vS1 vs. vS2, vS1 lesion, vS1 retro. inj.
17722	F	12	vS1/vS2	180 µm (1 sv.)	vS1: 2,865<br>vS2: 2,082	vS1 lesion, rad. eff.
18921	M	11	vS1/vS2	180 µm (1 sv.)	vS1: 3,277<br>vS2: 2,509	vS1 lesion, rad. eff.
14332	F	14	vS1/vS2	180 µm (1 sv.)	vS1: 3,454<br>vS2: 1,741	vS1 lesion, rad. eff.
18489	F	16	vS1/vS2	180 µm (1 sv.)	vS1: 2,493<br>vS2: 2,072	vS1 lesion, rad. eff.
18920	M	16	vS1/vS2	180 µm (1 sv.)	vS1: 2,904<br>vS2: 2,017	vS1 lesion, rad. eff.
20035	F	18	vS2	180 µm (2 sv.)	vS2: 4,138	vS1 retro. inj.
20428	M	14	vS2	240 µm (2 sv.)	vS2: 4,092	vS1 retro. inj.
20047	F	12	vS2	180 µm (2 sv.)	vS2: 3,157	vS1 retro. inj.
20044	M	12	vS2	240 µm (2 sv.)	vS2: 3,088	vS1 retro. inj.
19806	F	13	vS2	180 µm (2 sv.)	vS2: 2,940	vS1 retro. inj.
19821	F	23	vS2	180 µm (2 sv.)	vS2: 3,954	vS1 retro. inj.
16623	F	16	vS1/vS2	360 µm (2 sv.)	vS1: 4,868<br>vS2: 3,426	vS1 vs. vS2, vS2 lesion, sham lesion
14362	F	16	vS1	360 µm (2 sv.)	vS1: 5,159	vS2 retro. inj.
17517	M	17	vS1/vS2	360 µm (2 sv.)	vS1: 4,128<br>vS2: 3,301	vS1 vs. vS2, sham lesion
17721	F	21	vS1/vS2	180 µm (1 sv.)	vS1: 2,511<br>vS2: 1,440	vS2 lesion, sham lesion
18927	M	13	vS1/vS2	180 µm (1 sv.)	vS1: 2,676<br>vS2: 2,429	vS2 lesion, sham lesion
18933	M	13	vS1/vS2	180 µm (1 sv.)	vS1: 3,080<br>vS2: 1,905	vS2 lesion
20048	F	17	vS1/vS2	180 µm (1 sv.)	vS1: 2,720<br>vS2: 1,939	vS2 lesion
18922	M	17	vS1/vS2	180 µm (1 sv.)	vS1: 2,843<br>vS2: 2,205	vS2 lesion
18912	M	16	vS1	180 µm (2 sv.)	vS1: 5,136	vS2 retro. inj.
18919	M	16	vS1	240 µm (2 sv.)	vS1: 3,456	vS2 retro. inj.
20055	F	16	vS1	180 µm (2 sv.)	vS1: 5,123	vS2 retro. inj.
19781	M	18	vS1	240 µm (2 sv.)	vS1: 5,612	Sham lesion, vS2 retro. inj.

19770	M	15	vS1	180 µm (2 sv.)	vS1: 5,315	Sham lesion, vS2 retro. inj.
20421	M	13	vS1	180 µm (2 sv.)	vS1: 4,697	vS2 retro. inj.
20430	M	16	vS1	180 µm (2 sv.)	vS1: 3,559	vS2 retro. inj.
19776	M	23	vS1	240 µm (2 sv.)	vS1: 6,254	vS2 retro. inj.
16650	M	10	vS1/vS2	360 µm (2 sv.)	vS1: 5,191<br>vS2: 5,163	vS1 vs. vS2
16652	M	12	vS1/vS2	360 µm (2 sv.)	vS1: 3,146<br>vS2: 4,045	vS1 vs. vS2
14363	M	17	vS1/vS2	360 µm (2 sv.)	vS1: 4,141<br>vS2: 3,649	vS1 vs. vS2
17510	M	18	vS1/vS2	360 µm (2 sv.)	vS1: 5,644<br>vS2: 3,047	vS1 vs. vS2
17518	M	17	vS1/vS2	360 µm (2 sv.)	vS1: 5,160<br>vS2: 2,616	vS1 vs. vS2
14353	M	12	vS1/vS2	180 µm (1 sv.)	vS1: 2,804<br>vS2: 2,293	Sham lesion
14355	F	13	vS1/vS2	180 µm (1 sv.)	vS1: 2,315<br>vS2: 2,193	Rad. eff., vS1 retro. inj.
14354	F	13	vS1/vS2	180 µm (1 sv.)	vS1: 2,036<br>vS2: 1,467	Sham lesion, vS1 retro. inj.
14335	F	12	vS1/vS2	180 µm (1 sv.)	vS1: 2,839<br>vS2: 1,406	Sham lesion
18490	F	13	vS1/vS2	180 µm (1 sv.)	vS1: 3,032<br>vS2: 2,039	vS1 lesion, rad. eff.

Table S1, related to all Figures. Animal list. All mice were transgenic adult Ai162 X Slc17a7-Cre and therefore expressed GCaMP6s only in excitatory neurons. Spared whiskers were C2/C3 for all animals. We used a mix of male and female animals. Age of imaging onset is provided in weeks. Imaging scheme indicates the net depth span imaged across 1 or 2 subvolumes ('sv.'). Cell counts represent all neurons imaged per area and do not factor in restricted subregions (Methods). In general, each figure represents one key experiment and animals listed as part of that experiment comprise the dataset for each figure: vS1-vS2 comparison with broad depth sampling, 'vS1 vs. vS2' (**Figure 2**); lesion experiments (**Figures. 4, 5**). For retrograde injection animals ('retro. inj'; **Figure 3**), the injected area is specified; imaging took place in the other area. 'Rad. eff.' indicates animals included in the radius of effect analysis (**Figure S8**).

Ryan et al., Figure S1

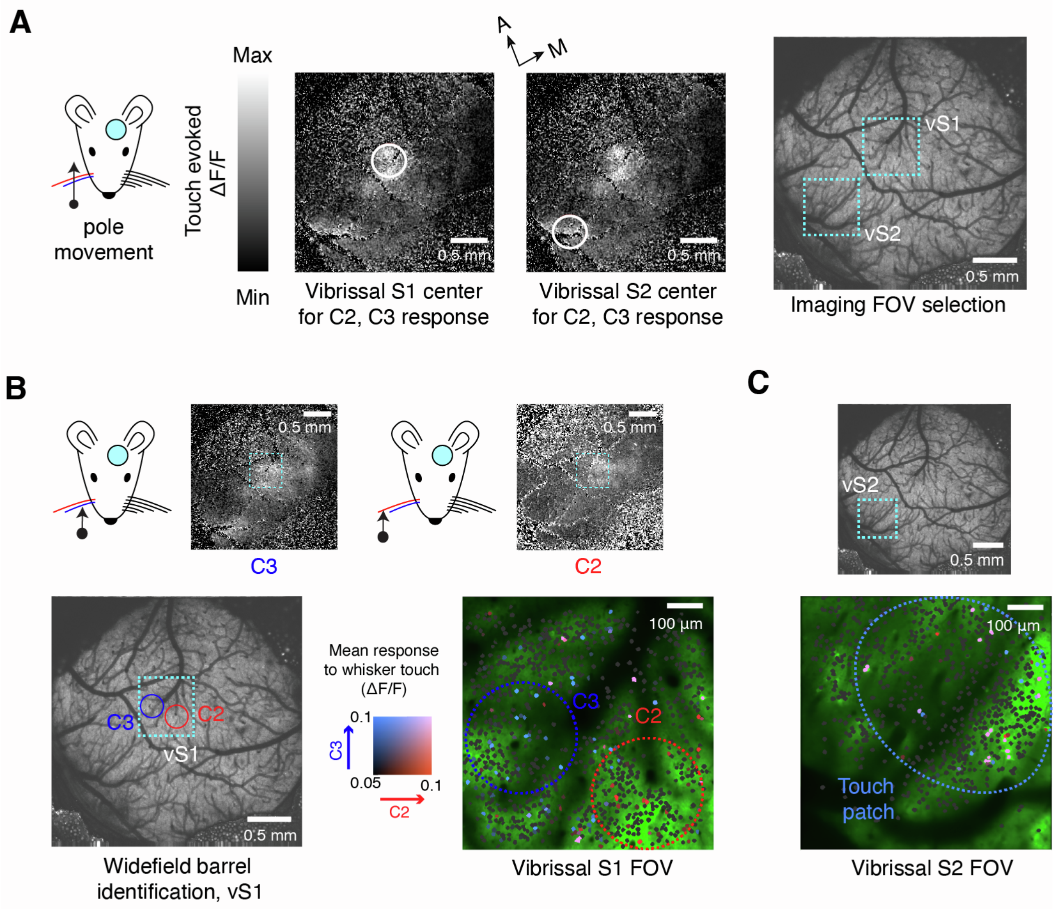

Figure S1, related to Figure 1. Vibrissal S1 barrel and vS2 touch patch identification.
A) Left to right; cartoon showing coarse stimulation of both spared whiskers at once via a pole pushing against the two whiskers; touch evoked widefield calcium response to such stimulation, with manually selected centers of vS1 and vS2 delimited in white; widefield 2-photon image of a cranial window with vS1 and vS2 fields of view outlined in cyan. Scale bar, 0.5 mm.
B) Top, same as in **A** but for stimulation of individual whiskers, revealing C3 and C2 barrel centers, again manually selected. Bottom, left to right: barrel locations noted within widefield image and vS1 imaging FOV; cellular resolution touch map within vS1 imaging FOV, with neurons colored by whisker preference and barrels overlaid. Scale bar, 100 µm.
C) Cellular-resolution touch-map for vS2 field of view in the same animal, with vS2 touch patch overlaid.

Ryan et al., Figure S2

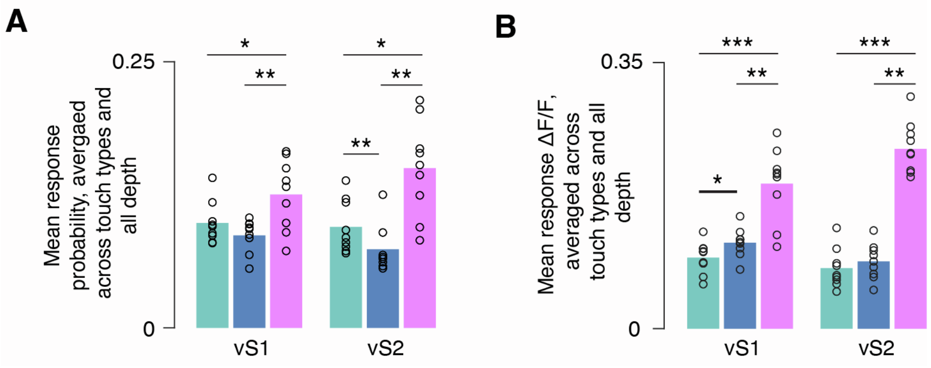

Figure S2, related to Figure 2. Contribution of specific touch cell types to touch response across all depths.

A) Mean response probability for each type in each area averaged across all touch types for which individual neurons were deemed responsive, including all sub-selected neurons across all depth (not restricted to superficial subvolumes as in **Figure 2E**).

B) Mean touch-evoked ΔF/F averaged across touch types for which individual neurons were deemed responsive, across all depths (not superficially restricted like **Figure 2F**). P-values indicated for paired t-test: * $p < 0.05$; ** $p < 0.01$; *** $p < 0.001$.

Ryan et al., Figure S3

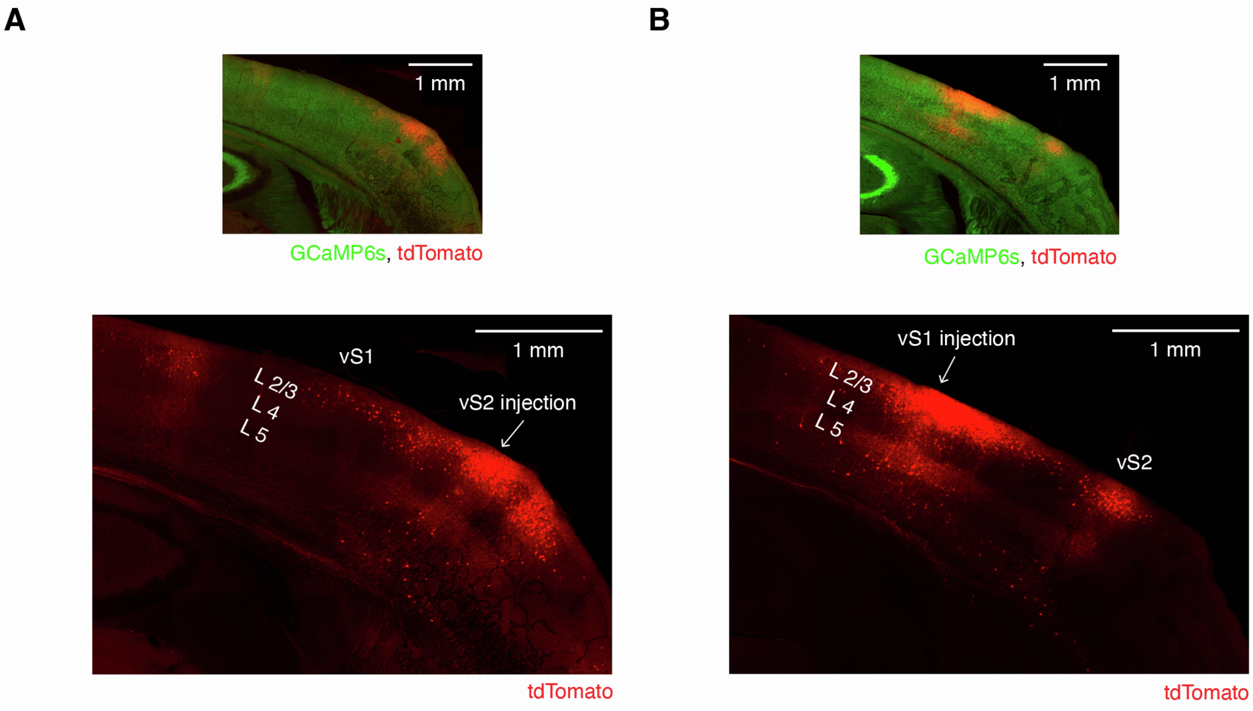

Figure S3, related to Figure 3. Distribution of retrogradely labeled neurons following viral injection into either vS2 or vS1.

A) Top: overlay of red and green channels from a confocal image displaying a retrograde injection in L2/3 of vS2 (Methods). Bottom: Red channel separated, 20x zoom. Injection site denoted. Scale bar, 1 mm

B) Same but for an injection in L2/3 of vS1.

Ryan et al., Figure S4

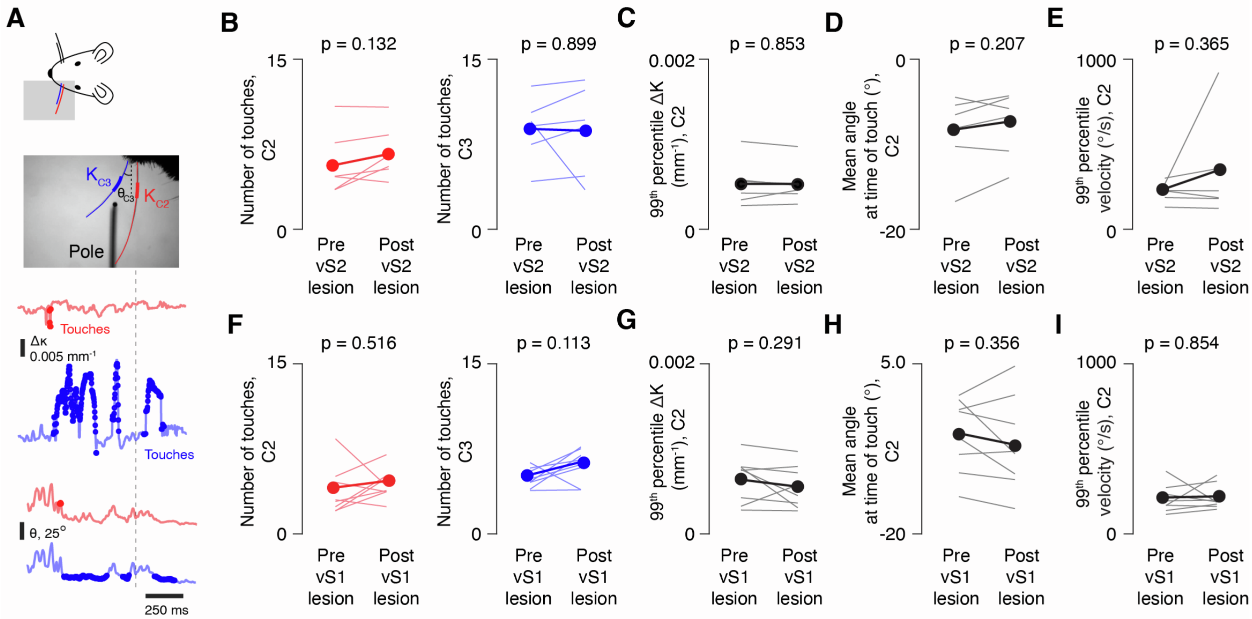

Figure S4, related to Figures 4 and 5. Lesions of vS1 and vS2 do not impact vibrissal kinematics.

A) Kinematic variables. Top, example whisker video frame, showing curvature (Δκ) measurement for both whiskers and angle (θ) measurement for C3. Bottom: Δκ and θ traces for both whiskers with touches overlaid.

B) Left (red), mean number of touches made by C2 across trials before and after vS2 lesion. Thick line: cross animal mean. Thin lines: individual mice, n=6. P-values indicated for two-sided paired t-test. Right (blue), same but for C3.

C) Peak change in curvature (Δκ) of C2 before and after vS2 lesion. 'Peak' is calculated by finding the 99th percentile of values across all whisker contact epochs.

D) Mean angle of C2 at the time of whisker touch before and after vS2 lesion, averaged across all times during which the pole was touched.

E) Peak velocity of C2 before and after vS2 lesion. Velocity is computed by comparing two subsequent frames (Methods), and 'peak' is calculated as the 99th percentile of values when the pole is in reach but prior to the first touch.

F-I) same as in **B-E** but comparing kinematics before and after vS1 lesions, n=8 mice.

Ryan et al., Figure S5

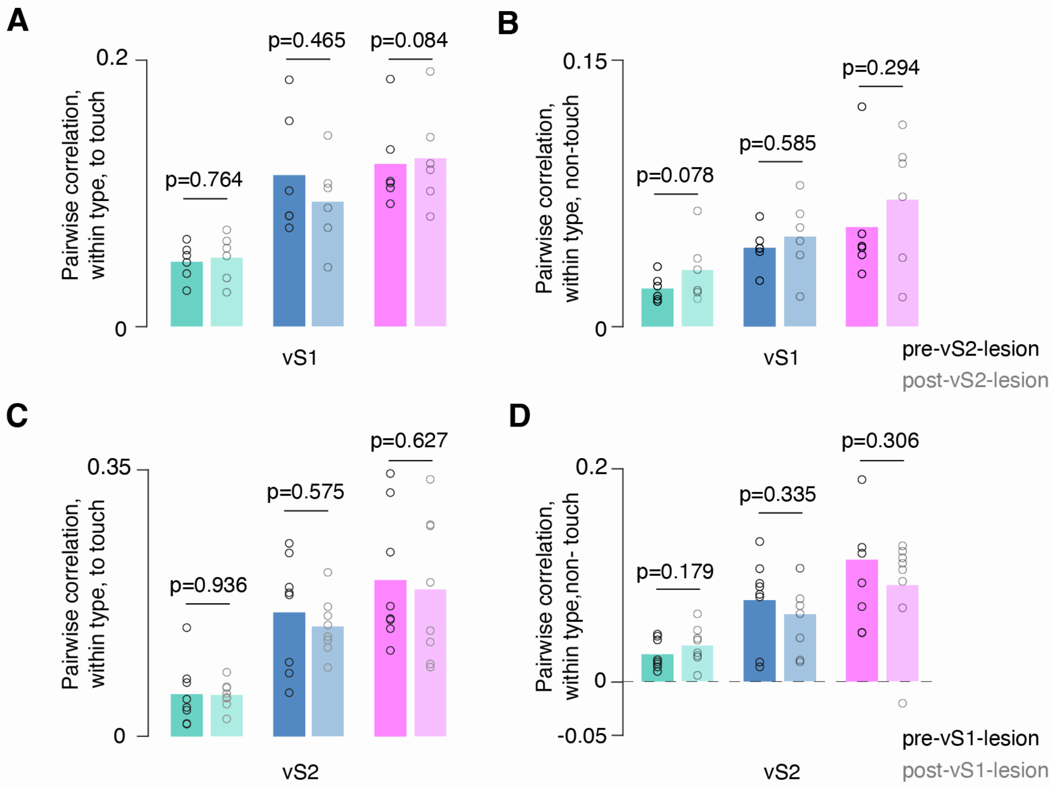

Figure S5, related to Figures 4 and 5. Columnar-scale lesions of vS1 and vS2 do not lead to overall changes in the correlation structure in the other area.

A) Mean within-type pairwise correlations in vS1 during the period around touch by group, pre-and post-vS2 lesion. Bars: cross- animal mean, circles: individual animal means. P-values are for two-sided paired t-test comparing mean correlation before and after vS2 lesions, n=6 mice.

B) Mean within-type pairwise 'spontaneous' correlations during non-touch period in vS1 before and after vS2 lesion.

C) Same as **A** but for vS2 before and after vS1 single barrel lesions; n=8 mice.

D) Same as in **B** for vS2 before and after vS1 lesion.

Ryan et al., Figure S6

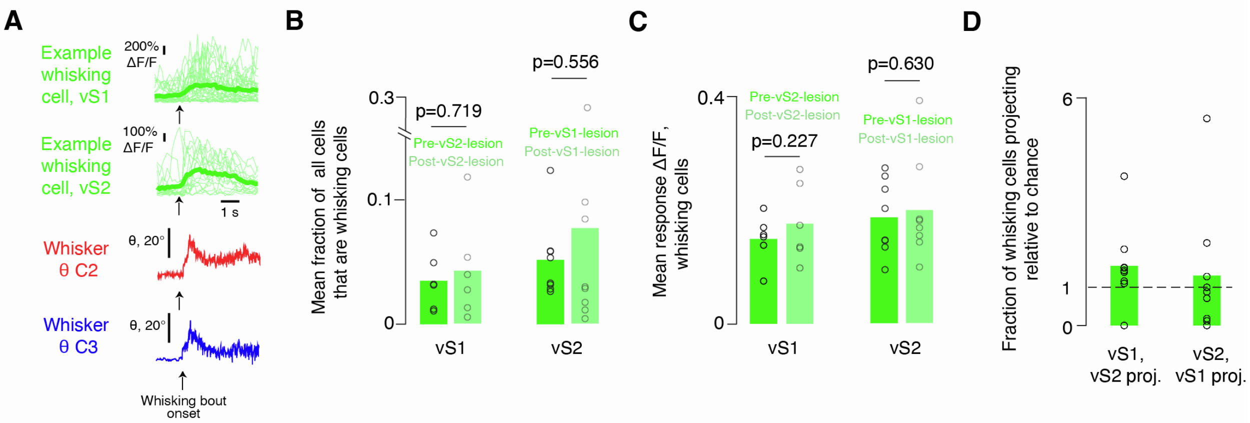

Figure S6, related to Figures 4 and 5. Columnar-scale lesions of vS1 and vS2 do not alter the whisking population in the other area.

A) ΔF/F traces for two example whisking cells, one from vS1 and one from vS2. Thin lines, individual trials; thick lines, mean. Mean angle traces for C2 and C3 shown below, all aligned to whisking bout onset.

B) Mean fraction of cells in each area that are responsive to whisking (Methods), before and after lesion.

C) Mean response ΔF/F of whisking cells before and after lesion in both areas.

D) Fraction of whisking neurons that project to the other area, normalized to fraction of all neurons that are whisking neurons (‘chance’).

Ryan et al., Figure S7

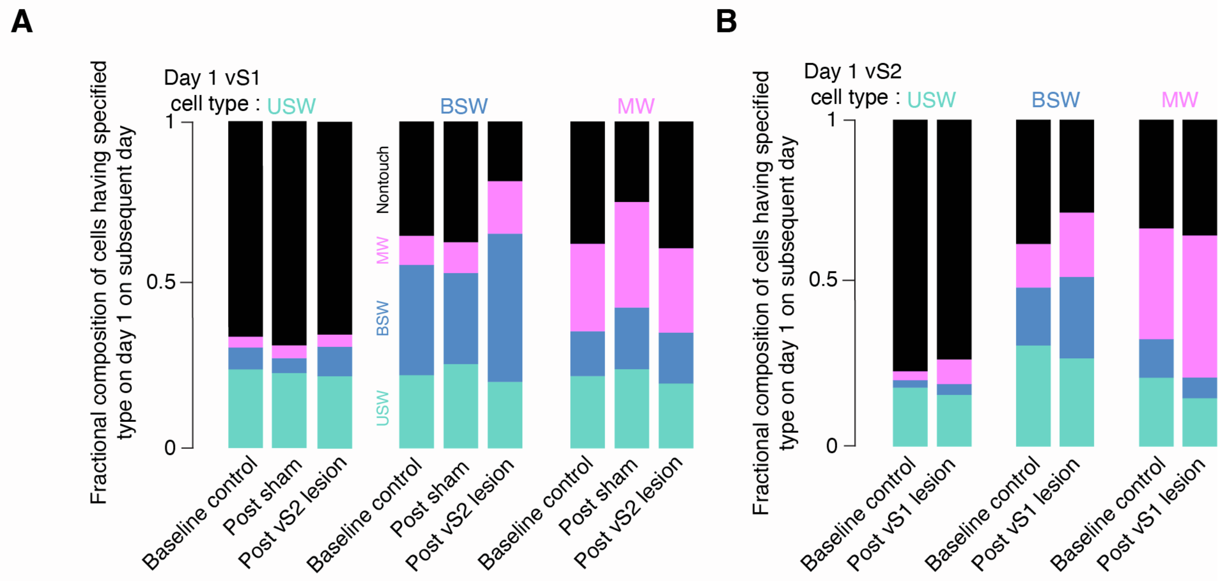

Figure S7, related to Figures 4 and 5. Change in touch neuron type on consecutive imaging sessions.

A) Touch type for neurons in vS1 on subsequent imaging day given their type on day 1, averaged across mice. For 'baseline control', day 1 is the first day of imaging, and day 2 is the second day of imaging, with no intervening perturbations (n=5). For the sham lesion, day 1 is the day before the sham lesion, and day 2 is 24 hours after the sham lesion (n=7). For the vS2 lesion, day 1 is the day prior to the lesion and day 2 is 24 hours after vS2 lesion (n=5). Black represents neurons that are not responsive to touch. There is no significant change in turnover rate for any cell type in the sham condition or the vS2 lesion condition.

B) Same as in **A** but for cells in vS2 (baseline control and vS1 lesion, n=6).

Ryan et al., Figure S8

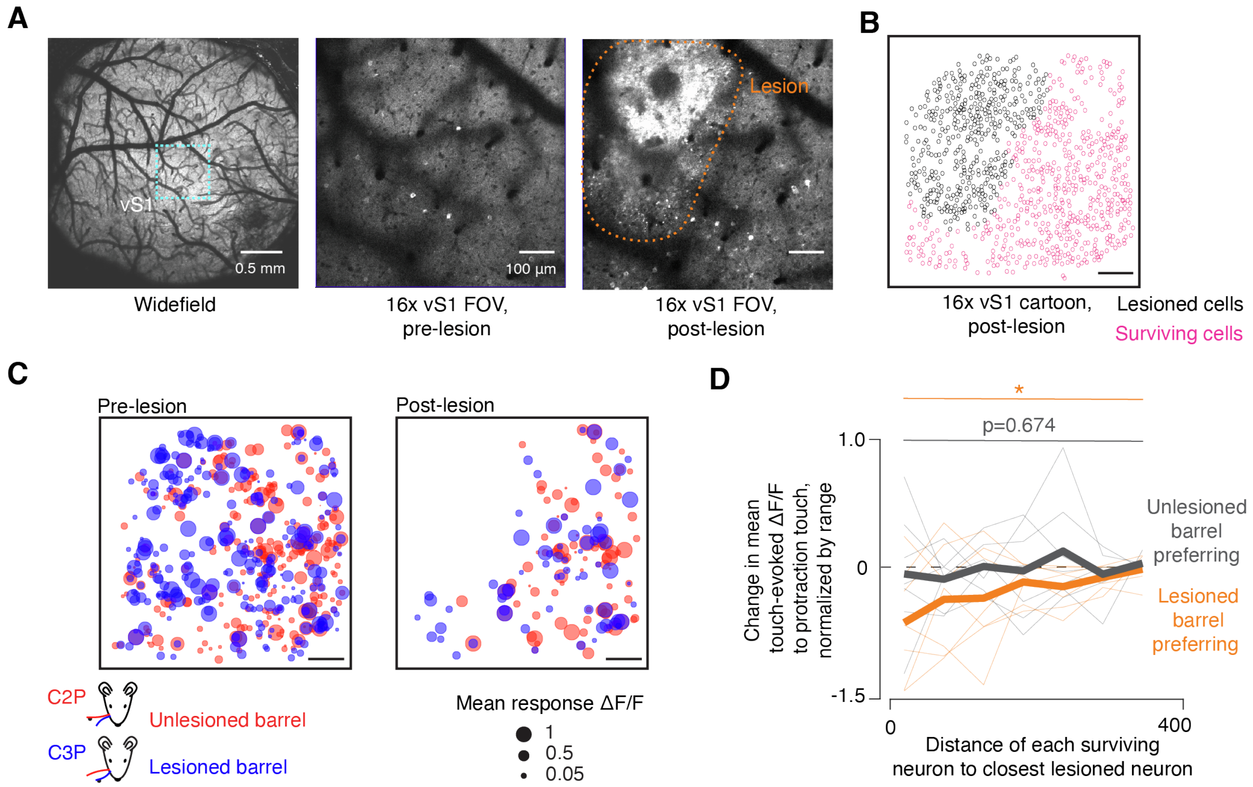

Figure S8, related to Figure 5. Radius of effect of single vS1 barrel lesion.

A) Left, vS1 field of view shown in widefield 2-photon image. Middle, vS1 imaging field of view at 16x. Right, same 16x field of view but after lesion to one barrel.

B) Cartoon of the same FOV showing which cells we determined to be dead after lesion (black) versus cells that survived (pink).

C) Map of touch-evoked ΔF/F for touch responsive neurons in vS1 to whisker C2 protraction (red) and whisker C3 protraction (blue) before (left) and after (right) lesion of the C3 barrel in vS1.

D) Normalized change in the mean protraction-touch-evoked ΔF/F as a function of each surviving neuron's distance to the closest lesioned neuron. Distance bins are 50 µm. Thin lines, mean change across neurons in a given distance bin for a single animal. Thick lines, cross animal mean. Orange, neurons that responded most strongly to whisker touch of the lesioned barrel's whisker. Grey, neurons that preferred to respond to the unlesioned barrel's whisker. P-values indicated for paired t-tests comparing distance bin 1 to the final distance bin for unlesioned and lesioned whisker responsive neurons; *, $p < 0.05$.

Ryan et al., Figure S9

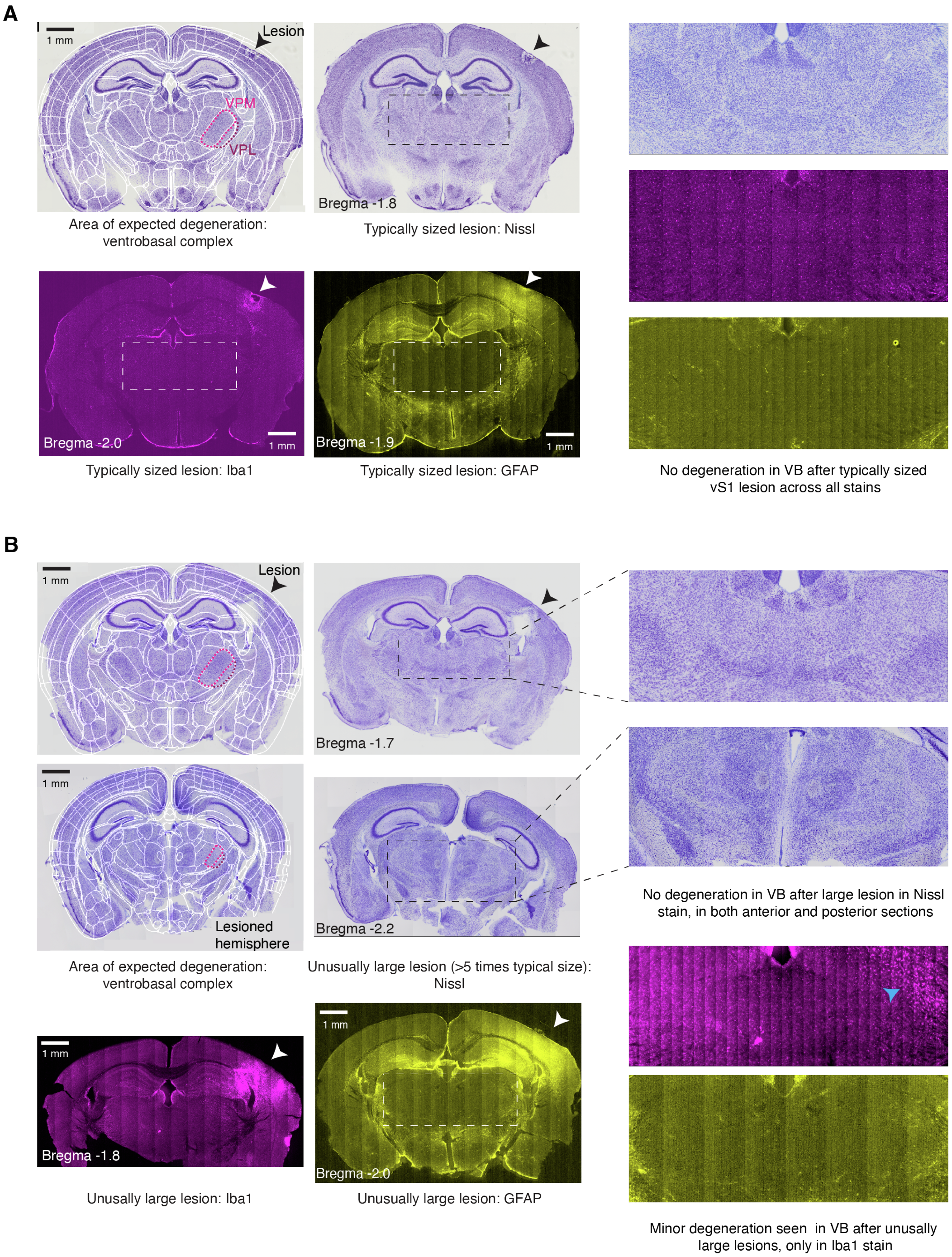

Figure S9, related to Figure 5. Impact of columnar-scale vS1 lesions on thalamus. A) Immunohistochemistry performed a few days after lesioning in example animals with normally sized lesions of vS1. Top left, Nissl stain, first overlaid with anatomical mapping from the Allen Brain Atlas, with thalamic areas in which we might expect degeneration (VB, including VPM and VL), outlined. Arrow shows lesion in vS1. Scale bar, 1 mm. Bottom left: Iba1 stained slice (magenta) and GFAP stained slice (yellow) with lesion

shown. Estimated AP distance from Bregma noted. Right: zoomed in view of all three slices, corresponding to outlined box on the left.

B) Same as in **A** but for example animal in which we made an intentionally large lesion, larger than 5x the size of our normal sized lesions (this lesion was not used for any experiments other than histology). Additional row of Nissl-stained images included from a more posterior section, as it is likely that the C2 and C3 barrels in vS1 project to an area of VPM that is slightly more posterior. Many of these lesions also went through the white matter. Blue arrow denotes possible thalamic degeneration.
